# Antibody targeting of anaerobic bacteria warms cold tumors and improves the abscopal effect of radiotherapy

**DOI:** 10.1186/s12967-024-05469-0

**Published:** 2024-07-15

**Authors:** WeiZhou Wang, YunXue Zheng, ZhouXue Wu, Min Wu, Yue Chen, Yan Zhang, ShaoZhi Fu, JingBo Wu

**Affiliations:** 1 Department of Oncology, The Affiliated Hospital of Southwest Medical University, Luzhou, Sichuan 646000 China; 2 Nuclear Medicine and Molecular Imaging Key Laboratory of Sichuan Province, Luzhou, Sichuan 646000 China; 3 Academician (Expert) Workstation of Sichuan Province, Luzhou, Sichuan 646000 China; 4 Department of Nuclear Medicine, The Affiliated Hospital of Southwest Medical University, Luzhou, Sichuan 646000 China; 5 Department of Oncology and Hematology, Affiliated Hospital of Traditional Chinese Medicine of Southwest Medical University, Luzhou, China

**Keywords:** *Bifidobacterium*, Monoclonal antibody, Tumor microenvironment, Hypo-fractionated radiotherapy, Immune checkpoint inhibitors, Abscopal effect

## Abstract

**Background:**

The combination of immune checkpoint inhibitors with radiotherapy can enhance the immunomodulation by RT and reduce the growth of distant unirradiated tumors (abscopal effect); however, the results are still not very satisfactory. Therefore, new treatment options are needed to enhance this effect. Our previous study showed that the combination of *Bifidobacterium* (Bi) and its specific monoclonal antibody (mAb) could target and alleviate hypoxia at the tumor site and act as a radiosensitizer. In this study, we explored the anti-tumor efficacy of quadruple therapy (Bi + mAb and RT + αPD-1). The current study also aimed to probe into the complex immune mechanisms underlying this phenomenon.

**Methods:**

Constructed 4T1 breast and CT26 colon cancer tumor models. A comprehensive picture of the impact of constructed quadruple therapy was provided by tumor volume measurements, survival analysis, PET/CT imaging, immune cell infiltration analysis and cytokine expression levels.

**Results:**

The abscopal effect was further amplified in the “cold” tumor model and prolonged survival in tumor-bearing mice. Bi can colonized in primary and secondary tumors and direct the mAb to reach the tumor site, activate complement, enhance the ADCC effect and initiate the innate immune response. Then combined with αPD-1 and radiotherapy to stimulate adaptive immune response and synergize with cytokines to expand the immune efficacy and generate effective anti-tumor immune response.

**Conclusions:**

Bi was used as an artificially implanted anaerobic target to cause a transient “infection” at the tumor, causing the tumor to become locally inflamed and “hot”, and at the same time, mAb was used to target Bi to enhance the local immune effect of the tumor, and then combined with radiotherapy and αPD-1 to amplify the abscopal effect in multiple dimensions. Therefore, the present study provided a new idea for the multipotent immune-activating function of antibody-targeted anaerobic bacteria for the RT treatment of extensively metastasized cancer patients.

**Supplementary Information:**

The online version contains supplementary material available at 10.1186/s12967-024-05469-0.

## Graphical abstract

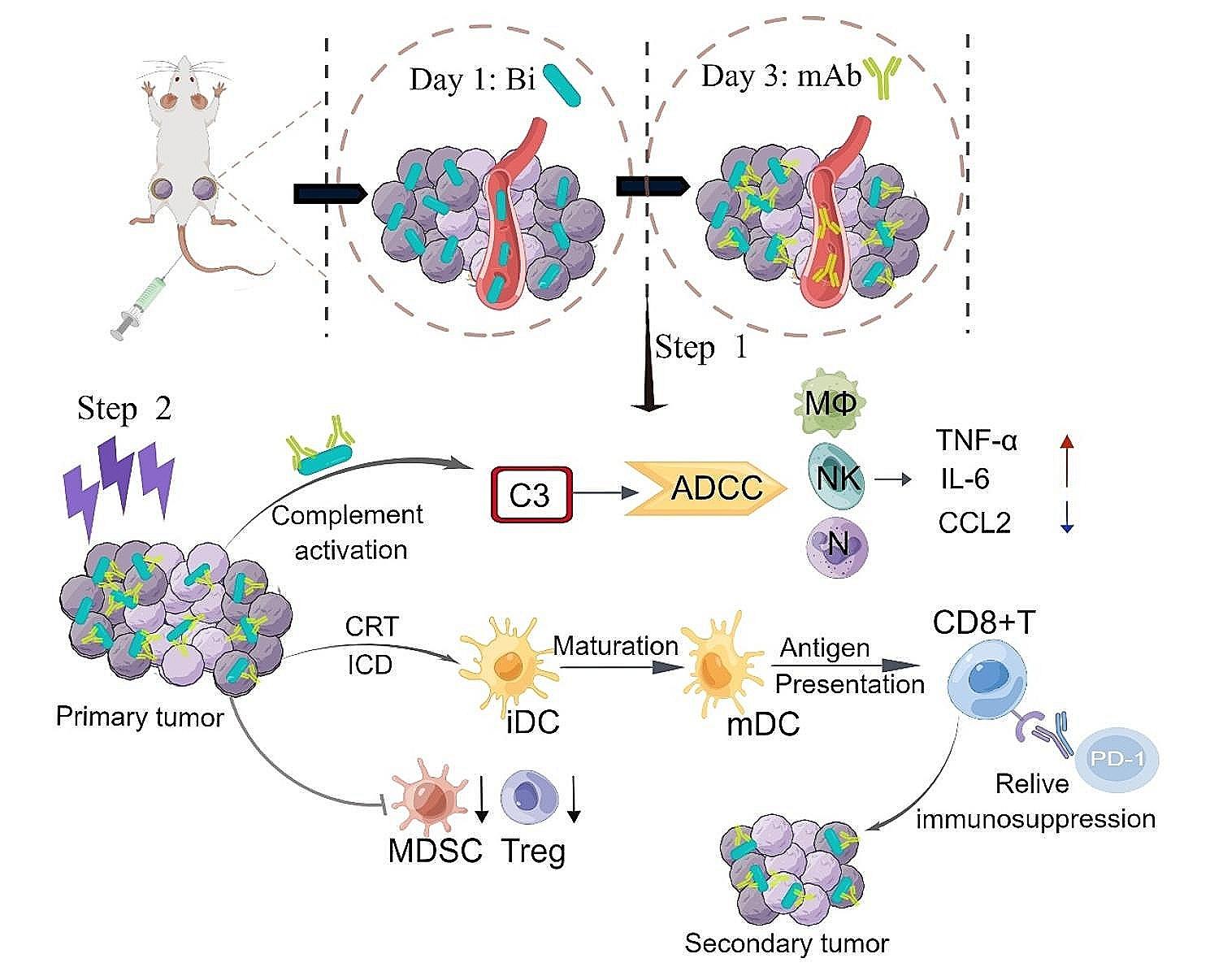

## Introduction

In 1953, R.H. Mole observed the shrinkage of non-irradiated tumor lesions after a patient received radiotherapy (RT) and proposed an abscopal effect [1]. However, the clinical incidence of RT-induced abscopal effects alone is extremely low [2]. Therefore, the effective induction of generating abscopal effect is a challenging question. Further research into the mechanism demonstrated that the abscopal effects are produced by activating the body’s immune system. RT causes immunogenic cell death (ICD), which exposes tumor antigens to become in situ tumor seedlings and induces the production of damage-associated molecular patterns (DAMPs), such as calreticulin (CRT). These DAMPs are expressed on the cell membrane surface and release the “eat me” signal [3, 4], promote the maturation of macrophages and dendritic cells (DCs), exercise their antigen-presenting function, and induce the activation of tumor-specific T-lymphocytes that migrate to unirradiated tumors and specifically recognize and kill the cells that are expressing similar tumor antigens to remove tumors in and out of the RT treated region [5, 6].

Numerous studies have shown that the RT dose and mode of division are important determinants of the antitumor immune response [7]. Hypo-fractionated radiotherapy (H-RT) improves the radiobiological effects and enhances the immunogenicity of the tumor. The increased dose of each RT session and decreased number of sessions, such as the administration of 8 Gy irradiation on 3 consecutive days (8 Gy × 3), is considered the most effective regimen for inducing abscopal effects and increasing the activity of cytotoxic T-lymphocytes [8, 9]. Studies have shown that the combination of RT and immune checkpoint inhibitors (ICIs) drugs can elevate the incidence of abscopal effects up to 20% [10, 11]. This is a significant but not very satisfactory improvement over the previous rate. Therefore, finding more effective ways to further improve this effect is an important subject to be studied at this time.

In recent years, drug-targeted delivery systems, including nano-biomaterials, have improved tumor targeting and therapeutic efficacy and have been used in a wide range of tumor therapies [12–15]. Among them, bacterial-mediated cancer therapy has returned to the limelight. This is due to the tendency of anaerobic bacteria to survive anaerobically, their ability to target hypoxic regions of tumors, and their capability to be used as an adjuvant for promoting both innate and adaptive immune responses [16–20]. In addition, bacteria can accumulate both in primary and secondary tumors; therefore, they can be used to treat metastatic tumors [21]. *Bifidobacterium* (Bi) is a specialized anaerobic immunopotentiator associated with a good response to anti-PD1 antibody (αPD-1) and has a good tissue-targeting ability. After intravenous administration, Bi was found to be distributed mainly in the hypoxic zone of tumors with very low toxicity and a good biosafety profile [22–24]. Based on the principle of antigen-antibody binding, we introduced Bi-specific antibody. We have demonstrated that Bi-specific antibodies could actively search and recognize Bi colonized in the tumor anoxic zone. And we further demonstrated that it could destroy the tumor anoxic zone, effectively inhibit the tumor micro-vessel generation, and reduce the anoxic condition in the tumors, thereby showing the effects of radio-sensitization [25]. Therefore, we hypothesized that Bi and its specific antibody could alter the tumor immune microenvironment from “cold” to “hot”, which would further enhance the abscopal effects of the combination of αPD-1 and Hypo-fractionated radiotherapy.

Based on this, a quadruple therapy model, consisting of Bi and its monoclonal antibody (mAb) combined with RT and αPD-1, was constructed in this study to observe its therapeutic efficacy on 4T1 breast cancer and CT26 colon cancer models in mice. Consequently, the therapy showed significant suppression in the growth of both primary and metastatic tumors and effectively induced the abscopal effects. In order to systematically delve into the immune mechanisms behind this quadruple therapy, the changes in the immune cell infiltration within the primary tumors, metastatic tumors, and spleen were evaluated. The results showed that Bi activated the complement system, enhanced ADCC action, and participated in the innate immune response after binding to its specific antibodies. The combination of Bi and its mAb with RT and αPD-1 promoted an adaptive immune response, relieved immune cells from their inhibitory state, and enhanced their recognition and killing of tumor cells, thus triggering a durable and safe antitumor immune response.

## Methods

### Cells, bacteria, antibodies, and animals

The 4T1 breast cancer cells and CT26 colon cancer cells were provided by the Cell Bank of Oncology laboratory, Southwest Medical University Hospital. Bi was purchased from the Guangdong Provincial Microbial Strain Preservation Center, and mAb was prepared by AbMax Biotechnology Co. (China) using commercial methods. Therapeutic αPD-1 mAb was purchased from Bio X cell. Six to eight-week-old female BALB/C mice were provided by Chongqing Tengxin Biotechnology Co. All the animal experiments were approved by the Experimental Animal Ethics Committee of Southwest Medical University.

### Tumor-bearing mice model and treatment

**Fig. 1:**
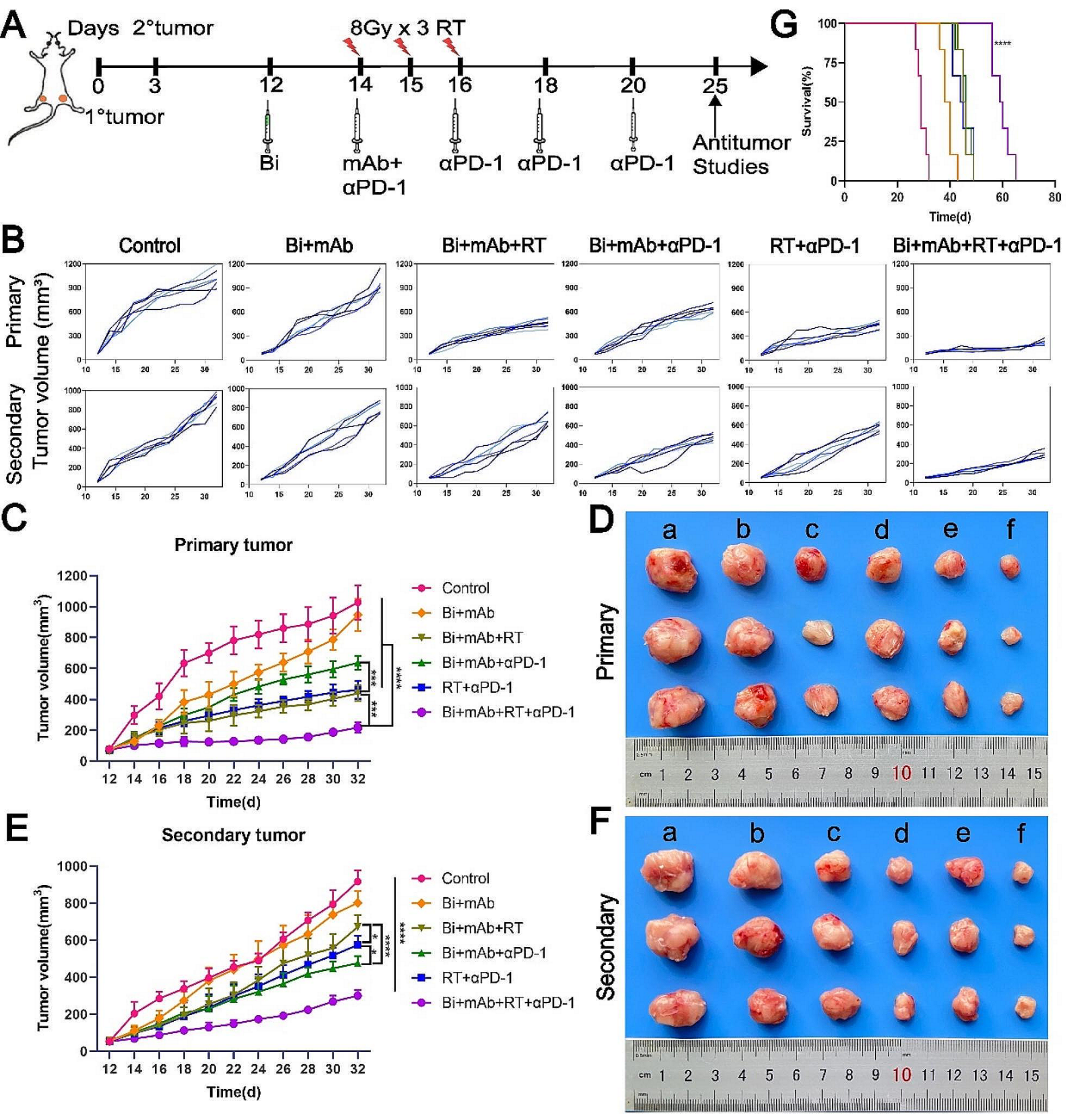
In vivo antitumor evaluation. (**A**) Treatment regimen to inhibit the growth of 4T1 tumors. Tumors that received irradiation were labeled as primary tumors (1°), while those that did not receive irradiation were labeled as secondary tumors (2°). (**B**) Growth curves of primary and secondary tumors in individual mice. Mean tumor growth curves of primary (**C**) and secondary (**E**) 4T1 tumors after treatment (*n* = 6). Images of stripped primary tumors (**D**) as well as secondary tumors (**F**) after euthanasia of mice on day 25: (**a**) Control, (**b**) Bi + mAb, (**c**) Bi + mAb + RT, (**d**) Bi + mAb + αPD-1, (**e**) RT + αPD-1, and (**f**) Bi + mAb + RT + αPD-1. (**G**) Survival of tumor-bearing mice (*n* = 6). All data are presented as the means ± SD. Tumor volumes on day 32 were statistically analyzed using one-way ANOVA. Mice survival rates were analyzed with the Kaplan– Meier method, and estimates were compared with log-rank (Mantel-Cox) tests. *P < 0.05, **P < 0.01, ***P < 0.001, ****P < 0.0001

The tumor cells were counted using a cell counting plate, and the density of the cell suspension was adjusted to 1.5 × 10^5^ 4T1 cells/0.1 mL or 2.5 × 10⁴ CT26 cells/0.1 mL. On day 0, 0.1 mL of the above cell suspension was injected percutaneously into the subcutis in the right thigh of each BALB/C mice. On day 3, the same amount of tumor cell suspension was injected into the subcutis in the left thigh of each mice. On day 12, when the primary tumor volume reached a size of 60–80 mm^3^ (volume = 0.5 × length × width²), it was treated after randomization into groups. On the same day, 0.2 mL (1.0 × 10⁷ cfu/mL) of Bi was injected intravenously into each mice, followed by the intravenous injection of 0.1 mL (0.15 mg/mL) mAb on day 14. Then, the mice were immobilized in a transparent box, and the primary tumors only in the right leg were irradiated locally at 8 Gy for three consecutive days (day 14, day 15, and day 16). Finally, αPD-1 (200 µg/injection) was injected intraperitoneally (Fig. 1A) on days 14, 16, 18, and 20, and the tumor volume was monitored every two days. The perpendicular diameter of each tumor was measured using Vernier calipers, and tumor volume was calculated using the following formula: length ×width² × 0.52, by two researcher independently. When mice were observed for survival, the mice were euthanized when the tumor volume exceeded 1100 mm³.

### Micro 18 F-FDG PET/CT imaging

In order to evaluate the early therapeutic effects of each treatment on tumor-bearing mice, images were taken on day 22 using a Micro PET/CT system (Siemens, Munich, Germany). The mice were fasted for more than 8 h (h), followed by the injection of 150–250 µCi 18 F-FDG into their tail vein. After 30 min, the mice were anesthetized with isoflurane inhalation, and PET/CT imaging was performed based on the following scanning parameters: voltage 80 kV, current 500 µA, layer thickness 1.5 mm, and 10 min per bed position. The acquired PET/CT images were then evaluated by two experienced nuclear medicine physicians to outline the region of interest (ROI) at each level of the tumor. Then, the maximum standardized uptake value (SUVmax) and mean uptake value (SUVmean) of the tumor tissue in the ROI were automatically calculated by the computer.

### Flow cytometry

On day 25, the mice were euthanized, and the tumor tissues on both the right and left sides of the mice were stripped followed by the removal of obvious surface necrotic tissues. The tissues were then gently sheared, and DMEM medium, containing 0.2% type IV collagenase, 0.01% hyaluronidase, and 0.002% DNaseI, was added to the sheared tumor tissues. On the other hand, the spleen tissues were peeled out and pounded into a homogenous pulp by physical milling, and then erythrocyte lysate was added to it. The tumor tissues were then placed in a water bath shaker at 37 °C and incubated with shaking for 35 min, while the spleen tissues were incubated under the same conditions for 15 min. The digestion enzyme digestion was terminated by adding fetal bovine serum. Finally, the digested tissue samples were centrifuged, washed with PBS, and filtered through a 200-mesh cell strainer to prepare a single-cell suspension for further analysis. Following the manufacturer’s instructions protocol (BD Biosciences, CA, USA), the obtained single cell suspensions were labeled with the following antibodies: CD4 + T cells and CD8 + T cells were labeled with CD45-PE-Cy7, CD3-APC, CD4-FITC, and CD8-PE; DCs were labeled with CD45-PE-Cy7, CD11b-APC, CD11c-PE, and CD86-FITC; Myeloid-derived suppressor cells (MDSC) cells were labeled with CD45-PE-Cy7, CD11b-FITC, and Gr1-APC; M2 cells were labeled with F4/80-PE, CD11b-FITC, and CD206-Alexa; N cells were labeled with CD45-PE-Cy7, CD11b-FITC, and Ly6G-PE; and NK cells were labeled with CD3-APC and CD49b-PE [26, 27]. The labeled samples were analyzed using a Beckman Coulter flow cytometric analyzer, and all the flow cytometry data were statistically analyzed using FlowJo software.

### ELISA

On day 25, mice were euthanized and orbital blood was taken and kept at room temperature for 45 min. The blood samples were then centrifuged at 2500 rpm for 20 min to obtain the upper serum layer. The tumor tissues were homogenized and ground, followed by centrifugation at 3000 rpm for 10 min, finally obtaining the supernatant. ELISA was performed for both the processed blood and tissue samples for C3, IL-6, IL-12, CCL2, TNF-α, IFN-γ.

### Measurement of lung nodules and histology

The mice’s lungs were removed on day 25 and fixed in 10% neutral buffer formalin for 24 h. The lung metastatic nodules were then counted. The fixed lung tissues were paraffinized, sectioned, and stained with H&E to assess intrapulmonary metastases.

### Bacterial biodistribution

When the tumor volume reached 60–80 mm³, 0.2 mL (1.0 × 10⁷ cfu/mL) Bi suspension was injected into each mice via the tail vein, and the mice were euthanized on days 1, 3 and 7 after injection. Then, the peripheral blood, left and right bilateral tumors, heart, liver, spleen, lung, and kidney tissues were homogenized, serially diluted, and plated on solid LB agar plates. All the plates were then cultured under anaerobic conditions at 37 °C for 48 h. The colony formation in the petri dishes was observed.

### In vivo imaging in mice

In order to assess the binding ability of mAb to Bi in vivo, mAb was labeled with indocyanine green (ICG), a near-infrared fluorescent dye. The mixture, containing 0.2 mg/mL ICG and 0.15 mg/mL mAb, was incubated for 24 h at room temperature. When the tumor volume reached 60–80 mm³, it was divided into three groups for imaging: ICG group (free ICG was injected on day 3); Bi + ICG group (Bi was injected on day 1 and free ICG was injected on day 3); and Bi + mAb@ICG group (Bi was injected on day 1 and ICG-labeled mAb was injected on day 3). After 6 h and 24 h of the fluorescent dye injection, the mice were anesthetized and imaged using the IVIS spectroscopy system. Finally, the major organs, including the heart, liver, spleen, lungs, and kidneys, as well as mice tumors were obtained for in vitro imaging.

### Immunohistochemistry and immunofluorescence

On day 25, mice were euthanized and all the sample tissues were fixed in 10% neutral buffered formalin, paraffinized, and then sectioned for immunohistochemistry and immunofluorescence. Following the manufacturer’s instructions, the tissue sections were labeled with the following antibodies: TUNEL, CD4, CD8, FOXP3, and CRT (Xavier Biotechnology Co., Ltd., Wuhan, China). Immunofluorescence images were acquired at the same brightness using CaseViewer. The percentage of positively stained cells was calculated using ImageJ software.

### Statistical analyses

We used GraphPad Prism Software, version 9 (GraphPad Software Inc.), and SPSS 27.0 software to conduct our statistical analyses. All data are presented as the mean ± SD. Data that were not normally distributed were transformed before analysis using square-root or log transformation. Unpaired *t* test was used for comparison between two groups. One-way analysis of variance (ANOVA) and Tukey post-hoc tests were used for multiple comparisons. Data that were not normally distributed after transformation were analyzed using the non-parametric Kruskal–Wallis test followed by Dunn’s multiple comparison test as a post hoc test. Survival curves were analyzed using the Kaplan-Meier method and compared with log-rank (Mantel-Cox) test. *P* values < 0.05 were considered statistically significant.

## Results

### Bi + mAb + RT + αPD-1 quadruple therapy inhibited primary and secondary tumor growth

The 4T1 breast cancer model is a solid tumor, which has the characteristics of spontaneous metastasis [28]. Therefore, Bi + mAb + RT + αPD-1 therapy was performed to treat 4T1 tumors to investigate whether this novel quadruple therapy model could effectively induce abscopal effects. When the primary tumor volume reached 60–80 mm^3^, the tumor-bearing mice were randomly divided into 6 groups, including Control, Bi + mAb, Bi + mAb + RT, Bi + mAb + αPD-1, RT + αPD-1, and Bi + mAb + RT + αPD-1, as shown in Fig. 1A. As compared to both the control and Bi + mAb groups, the mice primary tumor growth in all the other treatment groups was inhibited; the Bi + mAb + RT therapy more effectively inhibited the tumor growth, and the addition of PD-1 inhibitor further enhanced the inhibitory effects. On the contrary, for secondary tumors, Bi + mAb + RT did not show a significant reduction of tumor growth; however, Bi + mAb + αPD-1 treatment significantly reduced tumor growth and was superior to conventional RT + αPD-1 treatment. Finally, the quadruple therapy (Bi + mAb + RT + αPD-1) exhibited the greatest inhibitory effects on both the primary and secondary tumors as shown in Fig. 1B and F. In addition, it significantly prolonged the survival of tumor-bearing mice (median survival: 29 days in the control group, 29 days in the Bi + mAb group, 45.5 days in the Bi + mAb + RT group, 46 days in the Bi + mAb + αPD-1 group, 44.5 days in the RT + αPD-1 group, 59.5 days in the Bi + mAb + RT + αPD-1 group) as shown in Fig. 1G. In order to determine the dependency of Bi + mAb + RT + αPD-1 efficacy on the mice tumor type, another tumor model was established using mouse CT26 colon cancer cells and treated with the same treatment. The results showed that quadruple therapy more significantly inhibited the growth of the primary and secondary tumors (Supplementary Fig. S1), triggered distant effects, and exhibited a more significant survival benefit (Supplementary Fig. S2).

**Fig. 2:**
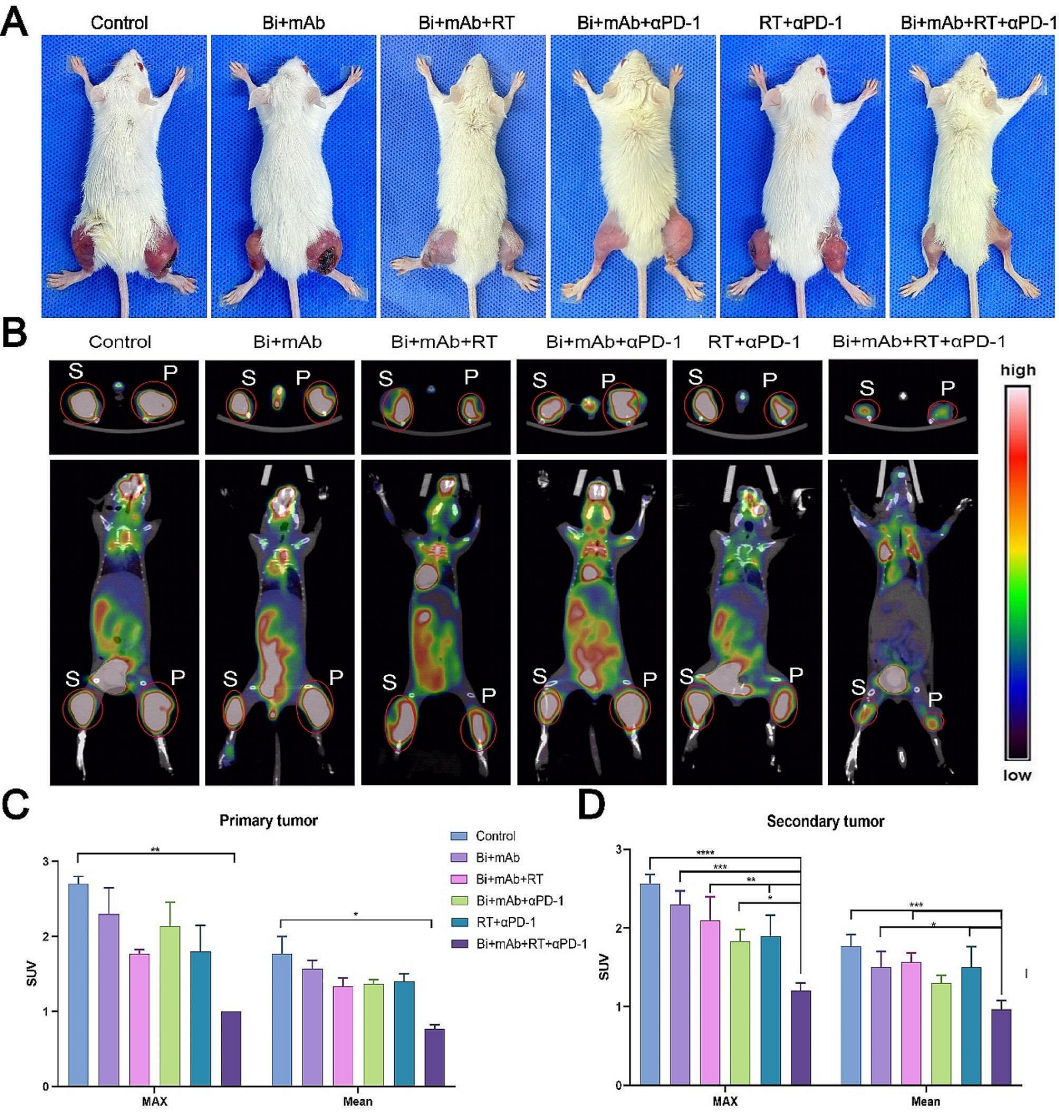
Micro-PET/CT imaging and gross view of tumor-bearing mice. (**A**) Representative images of tumor-bearing mice after treatment. (**B**) PET/CT images of 18 F-FDG in mice after treatment. The upper and lower parts are the cross-section of the tumor and coronal plane of the mice, respectively. SUVmax and SUVmean values after treatment of (**C**) primary and (**D**) secondary tumors (*n* = 3). All data are presented as the means ± SD. Statistical analyses were performed by Kruskal–Wallis test (**C**) and one-way ANOVA (**D**). *P < 0.05, **P < 0.01, ***P < 0.001, ****P < 0.0001

Gross images of tumor-bearing mice showed Bi + mAb + RT + αPD-1 treatment had minimal tumor volume (Fig. 2A). Furthermore, 18 F-FDG micro-PET/CT scans were performed on tumor-bearing mice to assess the early antitumor effects of various treatment modalities. The tumor cross-sections and coronal images showed that the Bi + mAb + RT + αPD-1 group exhibited the least tumor FDG uptake as shown in Fig. 2B. The ROI of the tumor site was outlined, and the FDG uptake values were quantitatively analyzed. The results showed that the SUVmax and SUVmean of primary and secondary tumors in the mice treated with Bi + mAb + RT + αPD-1 were much lower than those of the other groups. This indicated that the quadruple combination treatment effectively decreased the glucose metabolism level at the tumor site and significantly slowed down the tumor growth rate (Fig. 2C and D).

### Activation of the immune response by Bi and its specific mAb

Bi is a class of anaerobic bacteria. In order to study the biodistribution properties of Bi in vivo, Bi was injected into 4T1 tumor-bearing mice via their tail veins. On days 1, 3 and 7 days after injection, the mice were sacrificed, and their hearts, livers, spleens, lungs, and kidneys as well as their bilateral tumors were collected. The tissues were then homogenized, diluted, and plated on agar plates. It was found that on day 1, along with its distribution in tumor tissues, Bi was more distributed in the liver and lungs. On day 3, Bi was cleared from the main organs and mainly distributed in the bilateral tumors, showing better tumor targeting. Bacteria were also completely removed from the tumor by day 7. Interestingly, no bacteria were cultured in the blood, indicating that Bi had a good biosafety profile, excluding the occurrence of bacteremia (Supplementary Fig. S3).

Our previous study demonstrated that mAb could bind specifically to Bi [25]. In the present study, the specific binding of mAb to Bi was further verified using small animal in vivo imaging (IVIS). The imaging results showed fluorescent signals throughout the body after the injection of free ICG. On the other hand, a strong fluorescent signal was observed at the tumor site after 6 h of injection in the mAb@ICG group, and the signal was still observed after 24 h of injection (Supplementary Fig. S4A). It showed the active targeting ability of mAb on Bi. In addition, in order to be able to evaluate its biodistribution more clearly and intuitively, the main organs and tumor tissues of mice were taken for in vitro imaging (Supplementary Fig. S4B), and the imaging results showed that the mAb@ICG group had slight fluorescent signals in the liver, lung, and kidney, but more intense signals were manifested in the tumor tissues.

**Fig. 3:**
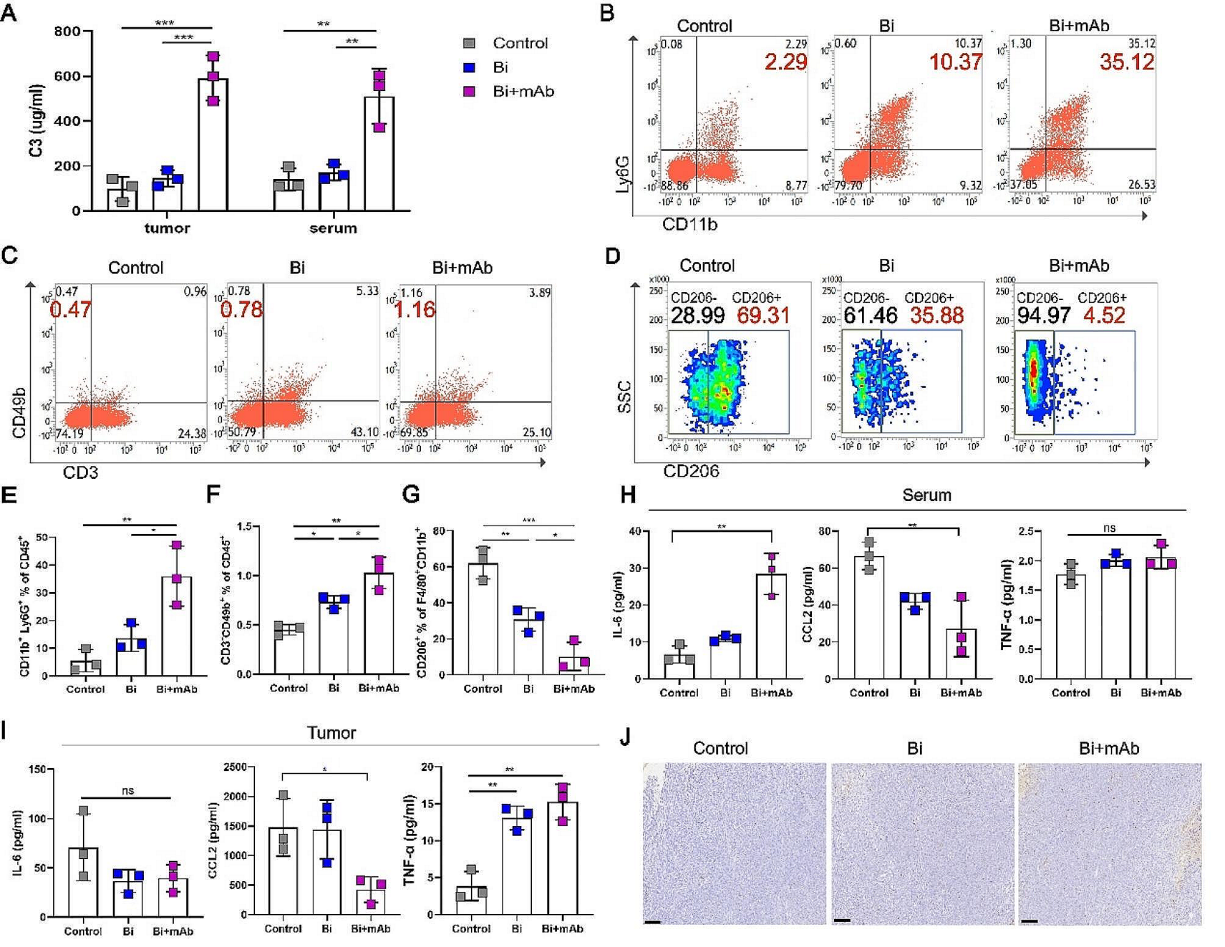
mAb activated the complement system and enhanced ADCC action. (**A**) Detection of C3 in tumor tissues and serum using ELISA. (**B** and **E**) Flow cytometry plots and proportion of neutrophils in tumor tissue. (**C** and **F**) Flow cytometry plots and proportion of NK cells in tumor tissue. (**D** and **G**) Flow cytometry plots and proportion of M2 macrophages in tumor tissue. (**H** and **I**) Expression of cytokines, such as IL-6, CCL2, and TNF-α in serum and tumor tissues. (**J**) Representative image of Tunel immunohistochemistry of tumor tissue. Scale bar, 100 μm. All data are presented as the means ± SD, *n* = 3. Statistical analyses were performed by one-way ANOVA and Kruskal–Wallis test. ns: not statistically significant, *P < 0.05, **P < 0.01, ***P < 0.001, ****P < 0.0001

The main mechanisms of action of the currently known antimicrobial mAbs are as follows: direct bactericidal effect; activation of NK, macrophages, neutrophils, and other immune cells FcR-mediated ADCC effects; complement-dependent cytotoxicity (CDC); and enhancement of cell phagocytosis [29, 30]. Therefore, to determine whether the binding of mAb to Bi could recruit complement components, mediate inflammation, enhance ADCC effects, and exhibit immune-dependent cytotoxic effects, the activation of the complement system was assessed after treatment with Bi and mAb, which indicated a significant elevation of tumor and serum C3 levels in Bi + mAb-treated mice (Fig. 3A). It is known that the activation of the complement system enhances the ADCC effect [31]. Therefore, the immune cell infiltration in the tumor was analyzed using flow cytometry. As compared to the control and Bi groups, Bi + mAb treatment increased the infiltration of neutrophils (Fig. 3B and E) and NK cells (Fig. 3C and F) into tumor tissue, while that of M2 macrophage was reduced (Fig. 3D and G). Furthermore, in order to assess Bi + mAb-mediated stimulation of systemic and local inflammatory responses, the serum and tumor levels of pro-inflammatory cytokines were determined. The Bi + mAb treatment increased the serum levels of IL-6 (Fig. 3H) as well as TNF-α levels at the tumor site (Fig. 3I), which mediated inflammation. The reduced levels of CCL2 were consistent with the downregulation of M2-type macrophage expression (Fig. 3H and I) [32]. Finally, a TUNEL assay was performed on tumor tissues to determine the antitumor effects of Bi + mAb treatment. The results demonstrated a higher level of tumor cell apoptosis after Bi + mAb treatment as compared to that in the control and Bi groups (Fig. 3J and Supplementary Fig. S9).

Overall, the specific binding of mAb to the Bi colonized in the tumor can be recognized by FcR-expressing killer cells, such as NK, macrophages, and neutrophils. Additionally, it can also trigger the ADCC effect, leading to the killing of nearby tumor cells because the killing effect of these cells is non-specific. mAb can also activate the complement system and enhance killer cell-mediated ADCC. mAb can participate in the innate immune response through immune cells (killer cells) and humoral immune molecules (complement); therefore, it can produce pro-inflammatory cytokines, especially TNF-α, which together induce apoptosis in tumor cells.

### Bi + mAb + RT + αPD-1 quadruple therapy increased antigen presentation

**Fig. 4:**
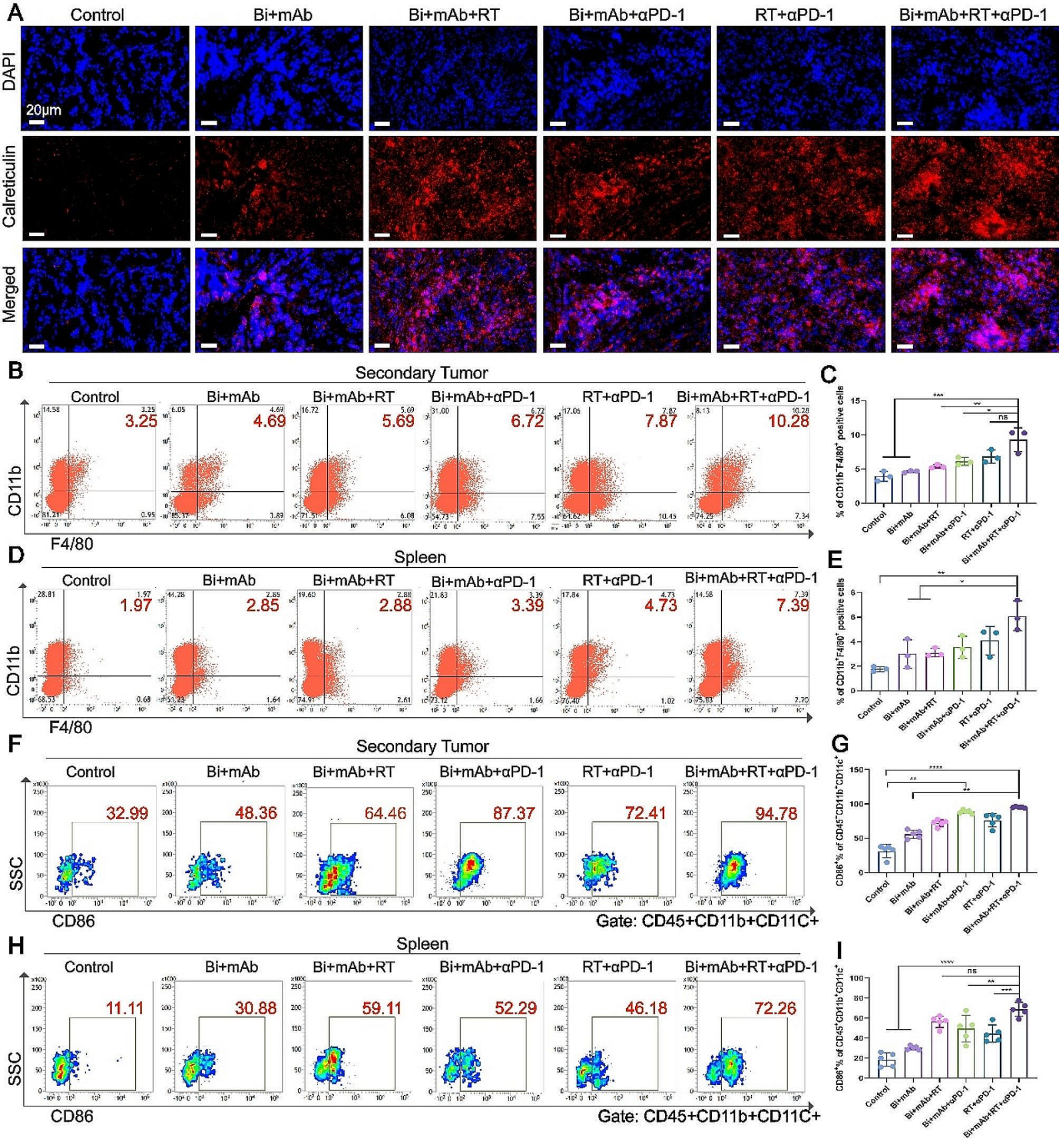
Exposure of calreticulin to attract antigen-presenting cells. (**A**) Representative immunofluorescence images of calreticulin in primary tumor tissue (*n* = 3). Scale bar, 20 μm. Flow cytometry dot plots and proportion of macrophages in the (**B** and **C**) secondary tumor and (**D** and **E**) spleen (*n* = 3). Flow cytometry dot plots and proportion of DCs in (**F** and **G**) secondary tumors and (**H** and **I**) spleen (*n* = 5). All data are presented as the means ± SD. Statistical analyses were performed by one-way ANOVA (**C**, **E**, and **I**) and Kruskal–Wallis test (**G**). ns: not statistically significant, *P < 0.05, **P < 0.01, ***P < 0.001, ****P < 0.0001

Bacteriotherapy and RT can induce the expression of DAMPs, such as CRT [4, 17], which acts as a mediator of immunogenic cell death and releases the “eat me” signals to antigen-presenting cells. Therefore, the effects of Bi + mAb and RT on the CRT expression in tumors were investigated. The immunofluorescence imaging showed a high expression of CRT in both the Bi + mAb + RT and Bi + mAb + RT + αPD-1-treated mouse tumors (Fig. 4A), indicating its potential role in the immune response.

Macrophages are highly phagocytic and can present phagocytosed antigens to T cells, thereby triggering an adaptive immune response [33]. In secondary tumors, the proportion of CD11b + F4/80 + macrophages was higher in the quadruple-treatment Bi + mAb + RT + αPD-1 group; the difference was insignificant as compared to that in the RT + αPD-1 group, while it was significant as compared to the other four groups (Fig. 4B and C). Meanwhile, in the spleen, the proportion of macrophages in the Bi + mAb + RT + αPD-1 group was significantly higher than that in the control, Bi + mAb, and Bi + mAb + RT groups (Fig. 4D, E).

DCs act as a linker between innate and adaptive immune responses, and mature DCs can upregulate the expression of CD86, which plays a key role in activating naive T cells and inducing antigen-specific T cell-mediated adaptive immune responses [34]. Among the secondary tumors, the proportion of CD86 + DCs was statistically upregulated in the Bi + mAb + RT + αPD-1 group compared to the control and Bi + mAb groups (Fig. 4F and G). In the primary tumors, the Bi + mAb + RT + αPD-1 group had significantly the highest proportion of CD86 + DCs (Supplementary Fig. S5). Meanwhile, in the spleen, the proportion of CD86 + DCs was the highest in the Bi + mAb + RT + αPD-1 group and also elevated in the Bi + mAb + RT group; however, the difference was not statistically significant (Fig. 4H and I). In conclusion, the Bi + mAb + RT + αPD-1 treatment increased the infiltration of macrophages and DCs in the tumor tissues and spleen, and promoted tumor antigen presentation.

### Bi + mAb + RT + αPD-1 quadruple therapy specifically increased the infiltration of CD8 + T cells in tumors

**Fig. 5:**
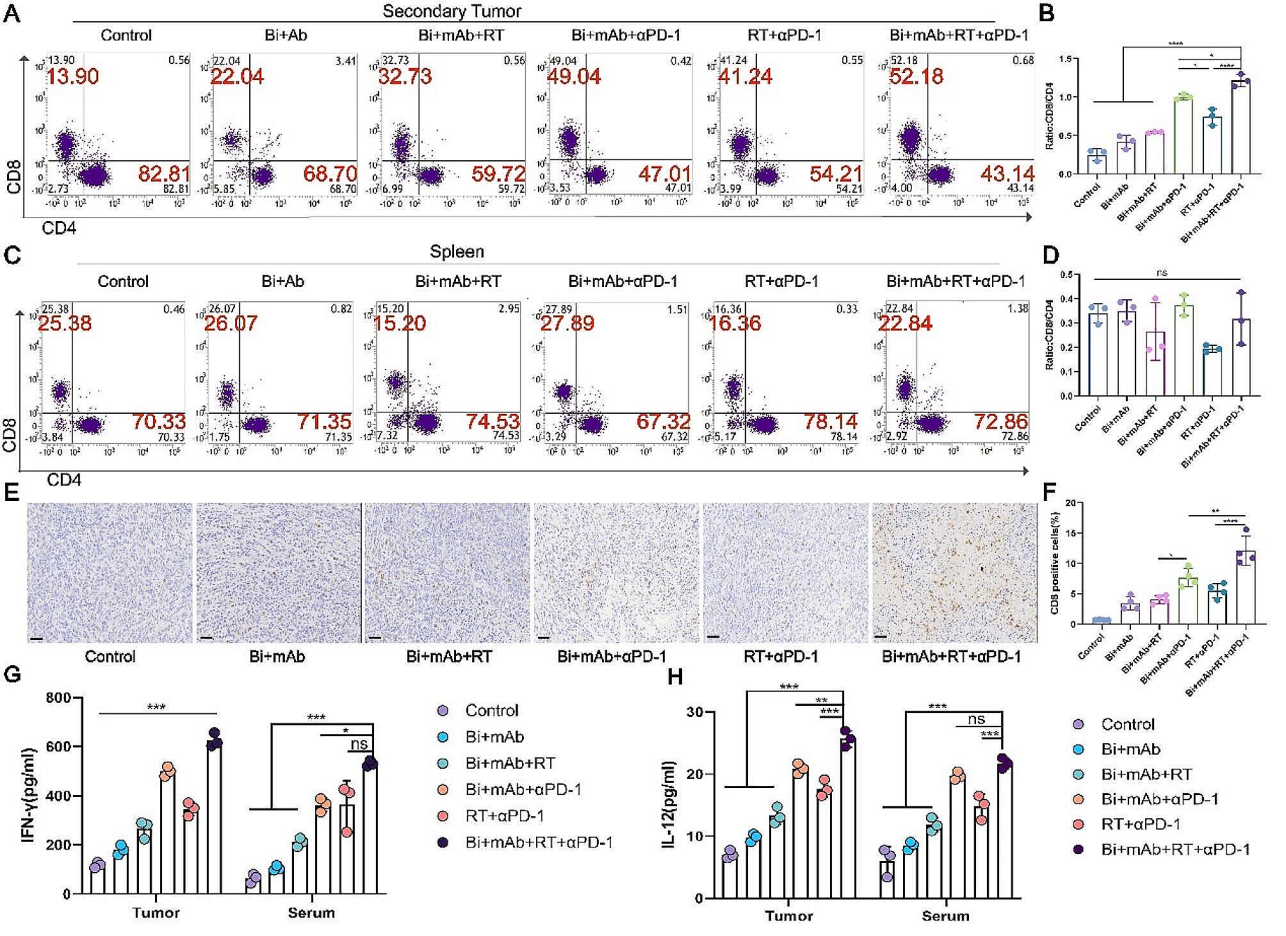
CD4 + and CD8 + T cell infiltration in mice under different treatments. Flow cytometry dot plots and CD8+/CD4 + T proportion of T cells in the (**A** and **B**) secondary tumor and (**C** and **D**) spleen (*n* = 3). (**E**) Representative images of CD8 immunohistochemistry in secondary tumors and (**F**) percentage of CD8 expression (*n* = 4). Scale bar, 50 μm. (**G** and **H**) Detection of IFN-γ and IL-12 in tumor tissue as well as serum by Elisa (*n* = 3). All data are presented as the means ± SD. Statistical analyses were performed by one-way ANOVA. ns: not statistically significant, *P < 0.05, **P < 0.01,***P < 0.001,****P < 0.0001

In order to determine how the increase in antigen-presenting cells activated the T-cells, the abundance of CD4 + and CD8 + T-cells in tumor tissues and spleen was investigated. In secondary tumors, Bi + mAb + αPD-1 treatment significantly increased the infiltration of CD8 + T cells as compared to that in the conventional RT + αPD-1 treatment group (Supplementary Fig. S10H). Moreover, the Bi + mAb + RT + αPD-1 group showed the highest CD8 + T-cell infiltration (Supplementary Fig. S10H) and a significant increase in the CD8 + to CD4 + T cell ratio (Fig. 5B). The CD8 immunohistochemistry also confirmed these results (Fig. 5E and F). In primary tumors, CD8 + T-cell infiltration increased after Bi + mAb + RT treatment, which made the tumors “hot” and sensitive to PD-1 inhibitors, and the proportion of CD8 + T-cells reached the highest proportion after the combined treatment of αPD-1 (Supplementary Fig. S6 and S10D). In the spleen, there was no difference in the number of T-cells among the treatment groups, which suggested that the CD8 + T-cell infiltration was tumor site-specific (Fig. 5C and D). The locally increased infiltration of CD8 + T-cells in the tumor promoted antitumor immunity and might be the key to the induction of abscopal effects.

Cytokines play an important role in the antitumor immune responses. Among them, the upregulation of IL-12 and IFN-γ expression levels promote T-cell infiltration and migration toward tumors, which in turn stimulates immunity, thereby playing a central role in the recognition and elimination of tumor cells [35, 36]. Therefore, the expression levels of these cytokines were measured in the tumor and serum. The results demonstrated that the overall Bi + mAb + RT + αPD-1 quadruple therapy increased IL-12 and IFN-γ secretion in serum as well as tumors (Fig. 5G and H). These cytokines can enhance both innate and adaptive immunity, contribute to the improvement of the immune response, and restrict the development and proliferation of the tumor.

### Bi + mAb + RT + αPD-1 treatment reversed the immunosuppressive tumor microenvironment

**Fig. 6:**
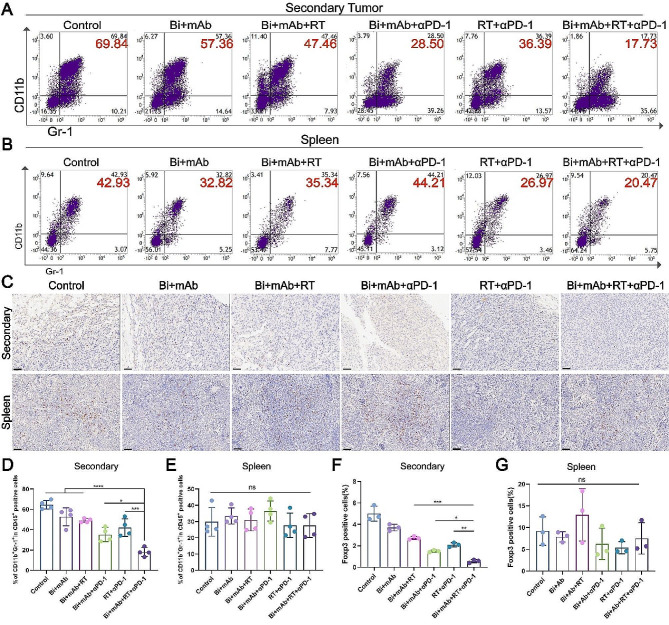
Infiltration of MDSCs and Treg cells in mice under different treatments. Flow cytometry dot plots and proportion of MDSCs in (**A** and **D**) secondary tumor and (**B** and **E**) spleen (*n* = 4). (**C**) Representative images of Foxp3 immunohistochemistry in secondary tumors and spleen. Scale bar, 50 μm. (**F** and **G**) Foxp3 expression levels in secondary tumors and spleens (*n* = 3). All data are presented as the means ± SD. Statistical analyses were performed by one-way ANOVA. ns: not statistically significant, *P < 0.05, **P < 0.01, ***P < 0.001, ****P < 0.0001

RT alone often cannot elicit an effective immune response, mainly due to the presence of an immunosuppressive microenvironment that allows tumor cells to escape immune surveillance. Myeloid-derived suppressor cells (MDSCs), a member of the tumor microenvironment, have a powerful immunosuppressive effect, and their expansion and accumulation can inhibit anti-tumor immune responses [37, 38]. Therefore, their abundance was examined using flow cytometry. In secondary tumors, MDSC infiltration was lower in the Bi + mAb + αPD-1 group than in the Bi + mAb + RT group and the RT + αPD-1 group, and the Bi + mAb + RT + αPD-1 group showed the lowest proportion of MDSCs (Fig. 6A and D). In primary tumors, the Bi + mAb + RT group did not significantly alter MDSC cell infiltration compared to that in the control and Bi + mAb groups, which decreased markedly along with reversal of immunosuppression after the addition of PD-1 inhibitors (Supplementary Fig. S7). In the spleen, there was also no difference observed in the proportion of MDSCs among the different groups (Fig. 6B and E). Foxp3, a Treg cell marker, can suppress effector T cell function and mediate immunosuppression [39]. Immunohistochemistry suggested that Bi + mAb + RT + αPD-1 quadruple therapy decreased the Foxp3 expression in both the secondary (Fig. 6C and F) and primary (Supplementary Fig. S8) tumors. In the spleen, Foxp3 expression was similar among all the treatment groups (Fig. 6C and G). Overall, the Bi + mAb + RT + αPD-1 treatment reduced the infiltration of MDSCs and Treg cells and remodeled the immunosuppressive microenvironment, thereby enhancing tumor cell clearance by effector T cells.

### Bi + mAb + RT + αPD-1 treatment effectively suppressed tumor and reduced lung metastasis

**Fig. 7:**
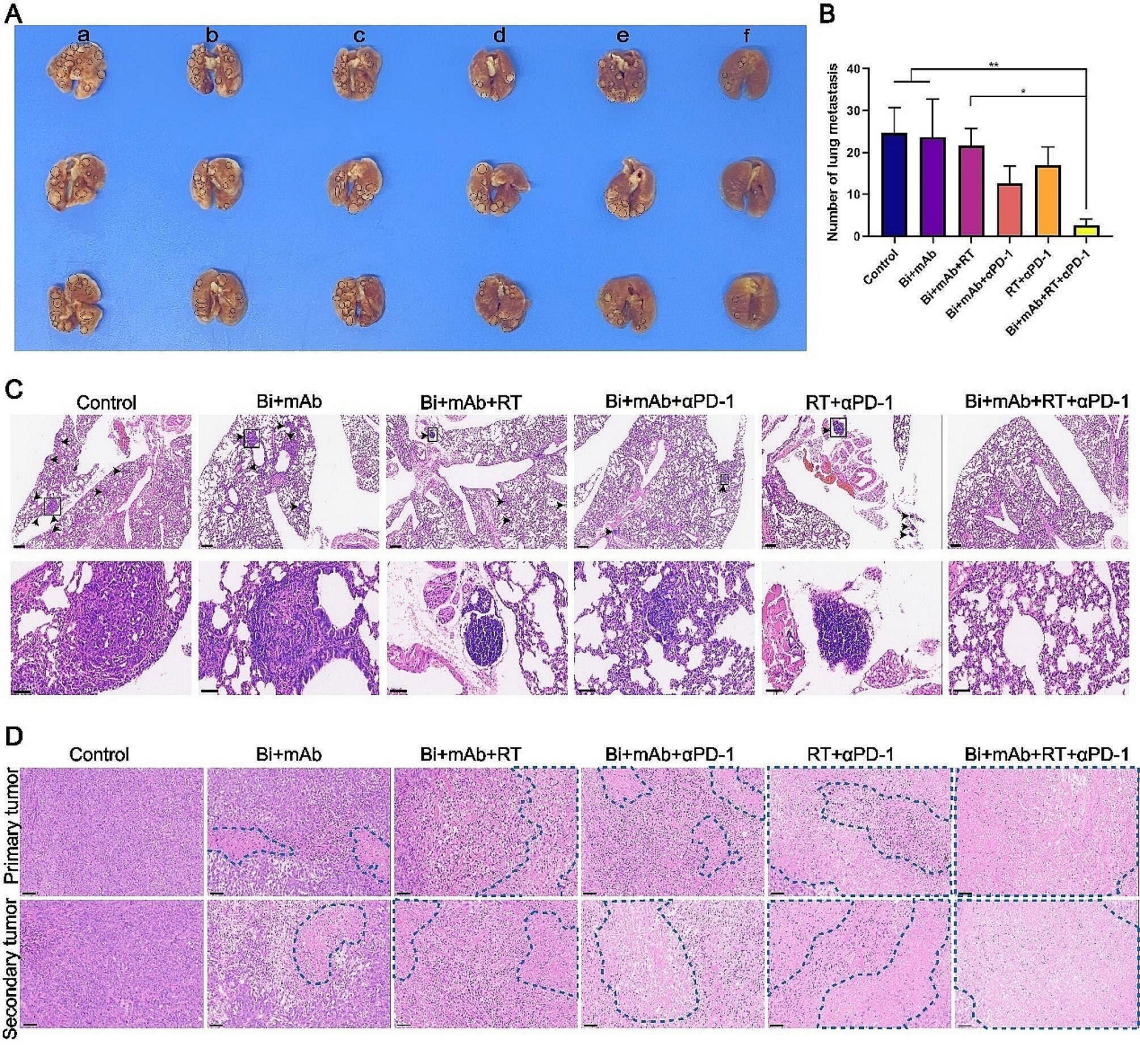
Bi + mAb + RT + αPD-1 quadruple therapy inhibited lung metastasis and accelerated tumor necrosis. (**A**) Macroscopic image of lung tissue and (**B**) statistical analysis of lung metastatic nodules (*n* = 3). (**a**: Control, **b**: Bi + mAb, c: Bi + mAb + RT, d: Bi + mAb + αPD-1, e: RT + αPD-1, f: Bi + mAb + RT + αPD-1). (**C**) H&E staining of lung tissue with metastatic lesions indicated by black arrows. Scale bars, 300 μm (upper layer) and 50 μm (lower layer, local magnification image). (**D**) H&E staining of tumor tissue; necrotic areas are circled by blue dotted lines. Scale bar, 50 μm. All data are presented as the means ± SD. Statistical analyses were performed by one-way ANOVA. *P < 0.05, **P < 0.01

Studies have shown that 4T1 tumor cells have spontaneous metastasis and often metastasize to the lungs. Therefore, the lung tissues were tested for metastatic nodules. The visible nodules on the surface of lung tissues were counted. The results showed that the Bi + mAb + RT + αPD-1 treatment group had a significantly lower number of metastatic nodules, indicating that quadruple therapy could effectively inhibit lung metastasis (Fig. 7A and B). The H&E staining of lung tissues showed that there were fewer metastatic clusters of tumor cells in the Bi + mAb + αPD-1 group as compared to those in the RT + αPD-1 group. Furthermore, no infiltration of tumor cells was observed in the sections of the Bi + mAb + RT + αPD-1 treatment group (Fig. 7C). In order to better visualize the tumor inhibition by each treatment, H&E staining was performed on primary and metastatic tumors. In primary tumors, Bi + mAb + RT and RT + αPD-1 treatments were more destructive, showing significant fragmentation and disappearance of cell nuclei. On the other hand, in secondary tumors, the Bi + mAb + αPD-1 and RT + αPD-1 groups showed more fragmentation and disappearance of cell nuclei in the tumor cells. The Bi + mAb + RT + αPD-1 treatment showed the best inhibition of both primary and secondary tumors, which could be identified in the microscopic observation of tumor cells, showing large swaths of death (Fig. 7D).

### Immuno-safety profile of Bi + mAb + RT + αPD-1 therapy

**Fig. 8:**
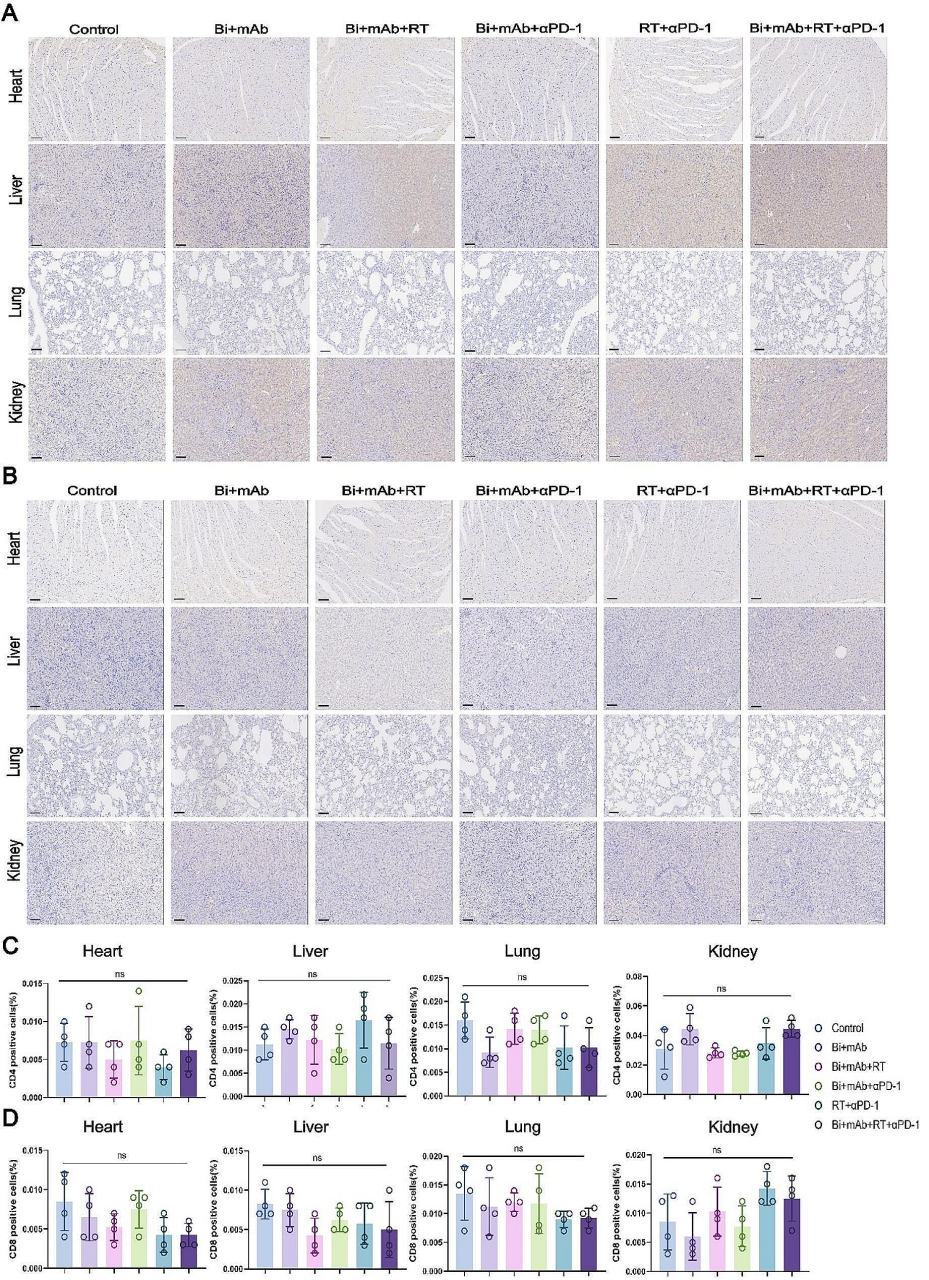
Infiltration of CD4 + and CD8 + T cells in major organs of mice. (**A**) Representative images for the CD4 immunohistochemistry of heart, liver, lung, and kidney and (**C**) percentage of CD4 expression (*n* = 4). (**B**) Representative images for the CD8 immunohistochemistry of heart, liver, lung, and kidney and (**D**) percentage of CD8 expression (*n* = 4). Scale bar, 100 μm. All data are presented as the means ± SD. Statistical analyses were performed by one-way ANOVA. ns: not statistically significant

Due to the distribution of PD-1/PD-L1 in normal organ tissues, one of the big questions about the application of ICI drugs is the possible occurrence of immune-related adverse events (IRAEs) [40], which might negatively affect the treatment. Therefore, immunohistochemistry for CD4 and CD8 was performed on the heart, liver, lung, and kidney tissues of mice (Fig. 8A and B). The results showed that the various treatments did not increase the infiltration of CD4 + and CD8 + T cells in normal organs (Fig. 8C and D). This suggested that despite some systemic immune activation, IRAE did not occur, and the treatment was safe.

## Discussion

Clinically, hypoxia poses significant difficulties in the RT of tumors, most notably in the form of resistance to RT [41]. In addition, hypoxia limits the immune cell infiltration, increases the infiltration of inhibitory cell populations, such as MDSCs and Treg, and suppresses the body’s antitumor immune response [42–44]. Therefore, the current study focused on alleviating tumor hypoxia, increasing immune cell infiltration, and reversing the immunosuppressive microenvironment in order to turn the “cold” tumors into “hot” tumors, leading to the amplification of RT-induced antitumor immunity. This study took advantage of the fact that Bi tends to survive anaerobically and can reach the tumor hypoxic zone, where it artificially implants a target. Based on the antigen-antibody reaction, Bi-specific mAb was prepared for targeting Bi. Unlike previous tumor therapeutic mAbs, the mAb used in the current study did not target tumor antigens, thereby avoiding the toxic effects on target antigens in normal tissues [45]. In addition, this mAb could actively bind specifically to Bi, colonized in tumors, thereby increasing the tumor site-specific accumulation of mAb (Supplementary Fig. S4). On the other hand, our previous study demonstrated that Bi, when combined with mAb, could effectively destroy the tumor’s hypoxic regions, inhibit the tumor micro-angiogenesis, improve the hypoxic condition of the tumor, and play the role of radio-sensitization in the tumor-bearing mice model [25]. The current study investigated the immune activation of mAb-targeted Bi as well as the immune mechanisms behind the enhanced abscopal effects of Bi + mAb in combination with RT + αPD-1 treatment in greater depth.

The abscopal effects of Bi + mAb + RT + αPD-1 quadruple therapy were observed in the 4T1 breast cancer and CT26 colon cancer models; this therapy effectively suppressed lung metastasis. The in-depth study of the mechanism of action showed that the inhibition of distant tumors was mainly achieved by the combined effects of innate and adaptive immune responses. First, the targeting of Bi by mAb increased the levels of C3 in tumor tissues and serum (Fig. 3A). Secondly, activation of the complement system enhances the killer cells (NK, macrophages, and neutrophils)-mediated ADCC effect (Fig. 3), resulting in non-specific killing of tumor cells. Lou’s study demonstrated that the increased IL-6 secretion indicated the extensive activation of the innate immune system [46]; this was consistent with the experimental results of the current study. In conclusion, mAb could bind to Bi and promote cellular and humoral immunity through killer cells and the complement system, which then together constituted the innate immune response. The innate immune response was further amplified by the increase in the levels of pro-inflammatory factors, such as IL-6 in serum and TNF-α in tumor tissues (Fig. 3I and J).

The low incidence of RT-induced distant effects is associated with the infiltration of immature DCs [47], which do not further activate T cells. In this study, the upregulation of CD86 on the surface of DCs in splenic and secondary tumors after Bi + mAb + RT + αPD-1 treatment effectively promoted the DC maturation and successfully induced the infiltration of CD8 + T-cells infiltration in the tumors (Figs. 4 and 5), thereby exhibiting significant inhibitory effects on primary and secondary tumors. Interestingly, there was no statistically significant difference in the number of T cell infiltration in the spleen, suggesting the tumor site-specific infiltration of CD8 + T cells. This showed that this treatment, despite low systemic immune activation, could promote a high local immune response at the tumor site. It was worth noting that the antitumor effects of Bi + mAb + αPD-1 therapy on secondary tumors were better than those of conventional RT + αPD-1 therapy, which might be because Bi and mAb could directly reach the distant tumors, increase the infiltration of local immune cells, convert the “cold” tumors into the “hot” tumors (sensitive to αPD-1), and loosen the immune “brakes” to further increase the cytotoxic T-cells. MDSCs and Treg cells, the major immunosuppressive cells, could inhibit CD8 + T cell function and weaken antitumor immune response. Bi + mAb + RT + αPD-1 quadruple therapy reduced the infiltration of MDSCs and Treg cells in secondary tumors, remodeled the immunosuppressive microenvironment, and facilitated the antitumor performance of CD8 + T cells.

In this study, Bi was injected intravenously. On day 3, there was almost no bacterial distribution in the normal tissues and organs, and it mostly accumulated in the bilateral tumor tissues. Compared to a previous study reporting that *Salmonella* VNP20009, a parthenogenetic anaerobe, had bacterial distribution in vital organs at day 10 [48], in the current study, the anaerobic Bi could better target tumors and was more biologically safe because it was completely cleared after treatment, and the mice did not develop bacteremia (Supplementary Fig. S3). Moreover, our previous study showed that Bi and mAb did not have significant toxic side effects on vital organs [25]. Due to the low induction of systemic immune activation by the treatment and its combination with a PD-1 inhibitor, the occurrence of IRAEs in the Bi + mAb + RT + αPD-1 group was assessed. As compared to the control group, the quadruple therapy did not increase CD4 + and CD8 + T-cell infiltration in vital organs and showed a lower likelihood of IRAEs. In addition, mAb, as a bacterial antibody, does not target tumor antigens, irrespective of the cancer’s genetic characteristics; therefore, it can be used for anaerobic bacteria-targeted therapy for a wide range of solid tumors. In addition, the Bi-mAb used in the current study were priced appropriately for production as compared to clinically used tumor therapeutic mAbs. Most importantly, mAb, as a stand-alone therapeutic drug, has been shown to mobilize killer cells to participate in the antitumor response. Moreover, combining mAb with nano-chemotherapeutic drugs or radionuclide drugs in order to improve drug targeting and reduce toxic side effects on normal tissues and organs, so as to achieve the precise delivery of drugs to the tumour site, obtaining the maximum therapeutic effect as well as the smallest side effects, optimizing the chemotherapy and radioimmunotherapy, which will be the focus of our follow-up study. Potentially, the antibody-targeted anaerobic bacteria strategy discussed in the current study might provide new ideas for amplifying the RT-induced distant tumor regression.

Clinical studies are somewhat lacking in comparison to the large number of animal models that have tested the immune and anti-tumor effects of various microbes. However, in contrast to the previous stagnation of microbiological clinical treatments, a growing number of researchers have now achieved breakthroughs in translating and applying discoveries from animal models to human treatments. For example, Dizman group evaluated the response of mRCC patients to the augmentation of ICI with CBM588, a *Bifidobacterium* product, which is currently in a phase I trial (NCT03829111). The results suggested that the patients treated with CBM588 achieved a higher objective response rate and a longer PFS [49]. There are also several clinical trials of microbiota combined with ICI underway, such as EDP1503, a *Bifidobacterium* product administered orally in combination with pembrolizumab for the treatment of triple-negative breast cancer [50]; and VE800, a drug consisting of 11 commensal organisms, in combination with nivolumab used to treat patients with advanced or metastatic cancer [51]. These clinical successes in the use of *Bifidobacteria* for cancer treatment have laid the foundation for the translation of our *Bifidobacterium* and its monoclonal antibodies from animal models to the clinic. In addition, we have made significant discoveries by combining bacterial therapy with immunotherapy and radiotherapy to treat cancer synergistically, which has broad application prospects and translational potential. However, there are corresponding limitations of concern: there are differences between mouse models and humans that may not fully replicate the human tumor microenvironment and the conduct of the immune response, and therefore efforts should continue to replicate the efficacy of quadruple therapy across a wider range of tumors, as well as in humanized mouse models, and ultimately in the clinical setting. It is also important to monitor whether the intestinal flora profile changes before and after treatment and the potential for clinical application with risks such as bacterial translocation. Although there are still challenges, bacteria have taken center stage in cancer immunotherapy, and combining bacterial therapy with radiotherapy, chemotherapy and immunotherapy is the way forward. At the same time, we can take advantage of the motility and proliferation of bacteria as drug carriers, and combine them with nanotechnology to improve drug targeting and reduce the toxicity and side effects of drugs on normal tissues and organs, thus achieving precise drug delivery at the tumor site, with maximum clinical efficacy and minimal side effects.

## Conclusions

In this study, a new quadruple therapy model (Bi + mAb + RT + αPD-1) was designed using Bi as a hypoxia-targeted bullet to direct the Bi-mAb to the tumor. The results showed that this anaerobic bacterium could bind to their antibodies and activate the innate immune response by promoting cellular and humoral immunity via killer cells and complement system, respectively. Moreover, the combination of bacterial treatment with RT induced ICD in tumor cells, increased the expression of mature DCs in the spleen and unirradiated tumors, which led to an increase in antigen presentation, promotion of CD8 + T-cell infiltration in the secondary tumors, and stimulation of the adaptive immune response. Furthermore, the quadruple therapy reduced the infiltration of MDSCs and Treg in the tumor tissues and reversed the immunosuppressive microenvironment for the killing effect of CD8 + T cells. Thus, multiple dimensions of tumor transformation into an immunologically responsive “hot” state as well as synergistic innate and adaptive immune responses were achieved for the tumor killing.

### Electronic supplementary material

Below is the link to the electronic supplementary material.

Supplementary Material 1

Supplementary Material 2

## Data Availability

The datasets used and/or analyzed during the current study are available from the corresponding author on reasonable request.
