# Supplementary material for: Antibody targeting of anaerobic bacteria warms cold tumors and improves the abscopal effect of radiotherapy
Source: J Transl Med. 2024 Jul 15;22:657. doi: 10.1186/s12967-024-05469-0 (PMC11247849; doi:10.1186/s12967-024-05469-0)
Supplement: Supplementary file 1 — Supplementary Material 1 [file 12967_2024_5469_MOESM1_ESM.docx]

**Supporting Information**

**
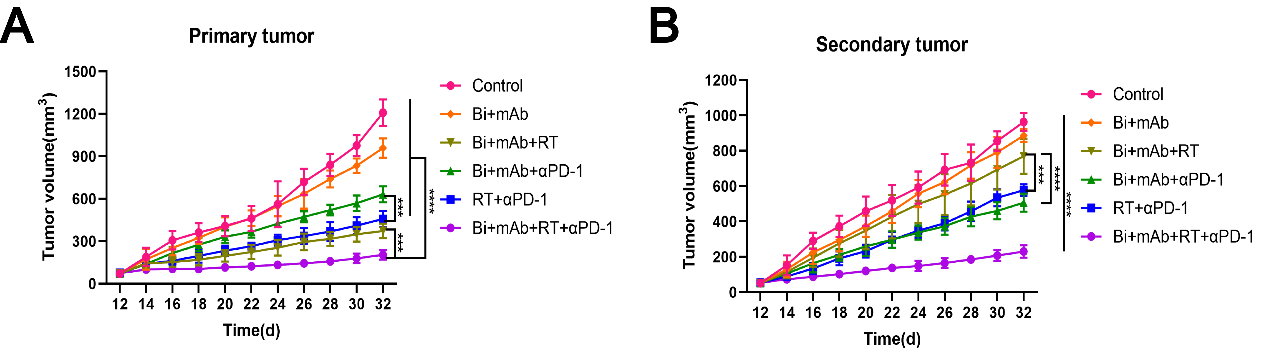
Figure S1.** *In vivo* anti-tumor evaluation of CT26 colon cancer. Mean tumor growth curves after treatment of primary (A) and secondary (B) CT26 tumors (n=6). All data are presented as the means ± SD. Tumor volumes on day 32 were statistically analyzed using one-way ANOVA. ***P<0.001, ****P<0.0001.


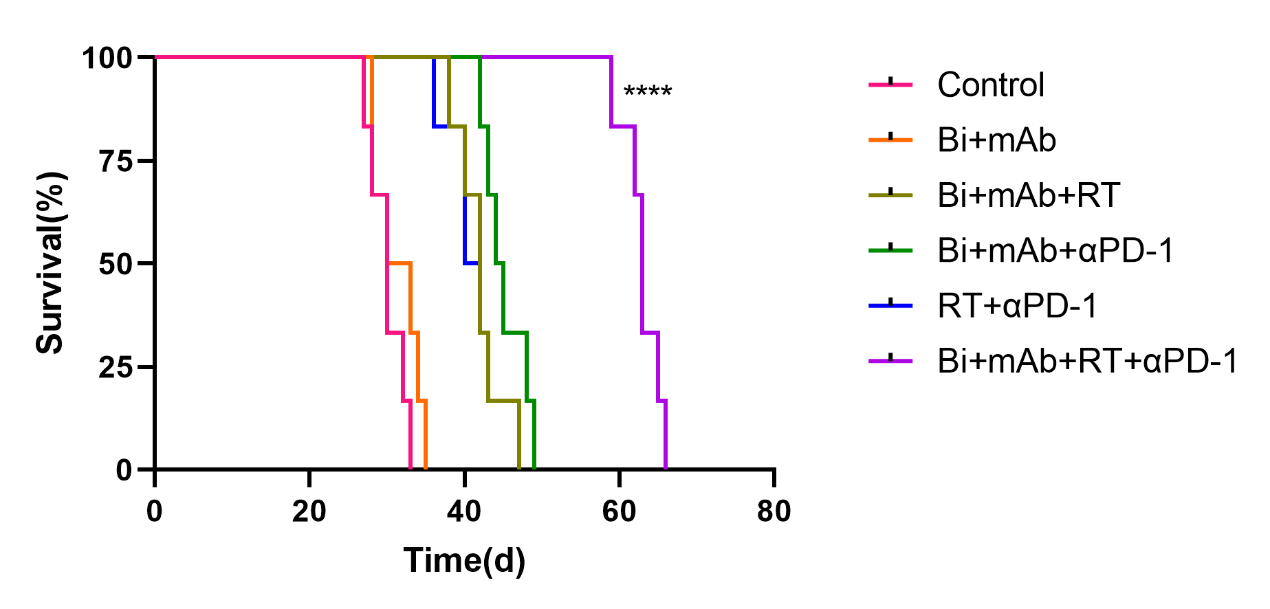


**Figure S2.** Survival of tumor-bearing mice (n=6). Mice survival rates were analyzed with the Kaplan– Meier method, and estimates were compared with log-rank (Mantel-Cox) tests. ****P<0.0001.


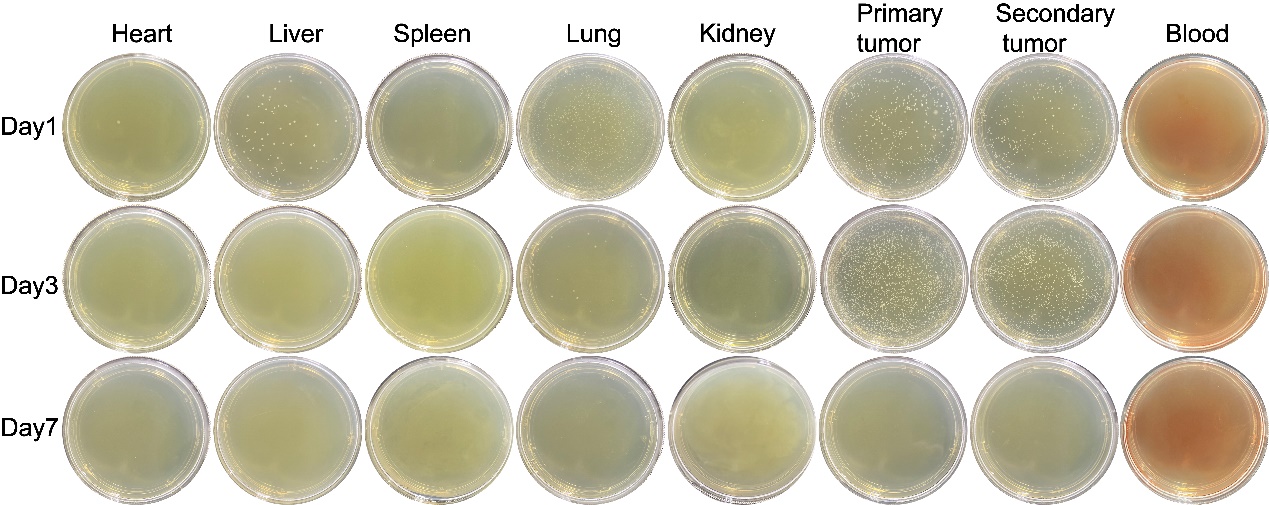


**Figure S3.** Distribution of bacteria in the heart, liver, spleen, lungs, kidneys, bilateral tumors and in the blood on days 1, 3 and 7 after bacterial injection.


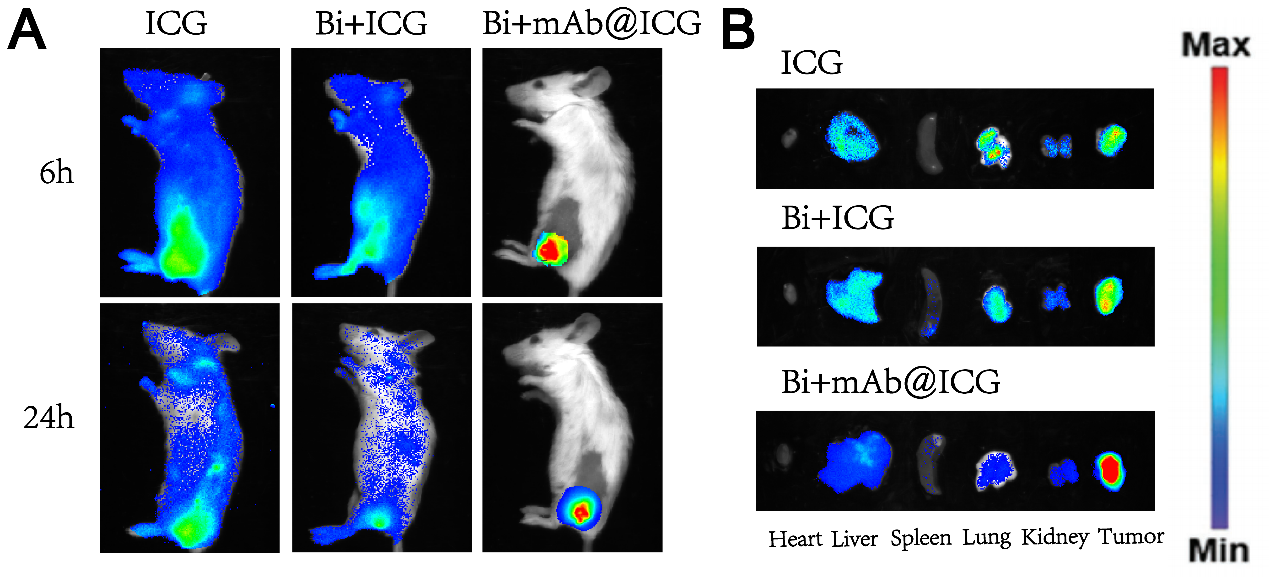


**Figure S4.** The mAb was biodistributed in vivo. It was divided into 3 groups: ICG group (free ICG was injected on day 3); Bi+ICG group (Bi was injected on day 1 and free ICG was injected on day 3); and Bi+mAb@ICG group (Bi was injected on day 1 and ICG-labeled mAb was injected on day 3). (A) IVIS imaging was performed 6h and 24h after ICG or mAb@ICG injection. (B) In vitro fluorescence images of isolated organs and tumors at 24 h of post injection.


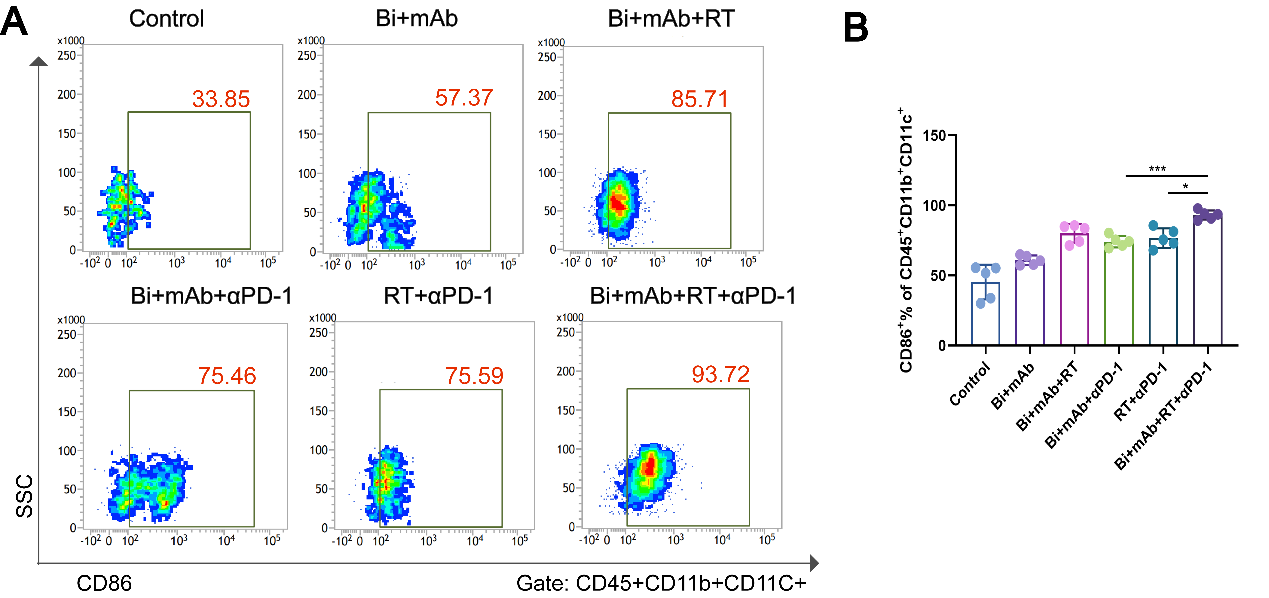


**Figure S5.** Dendritic cell infiltration in mice under different treatments. (A) Flow dot plot of dendritic cells in the primary tumor and (B) percentage (n=5). All data are presented as the means ± SD. Statistical analyses were performed by one-way ANOVA. *P<0.05, **P<0.01.


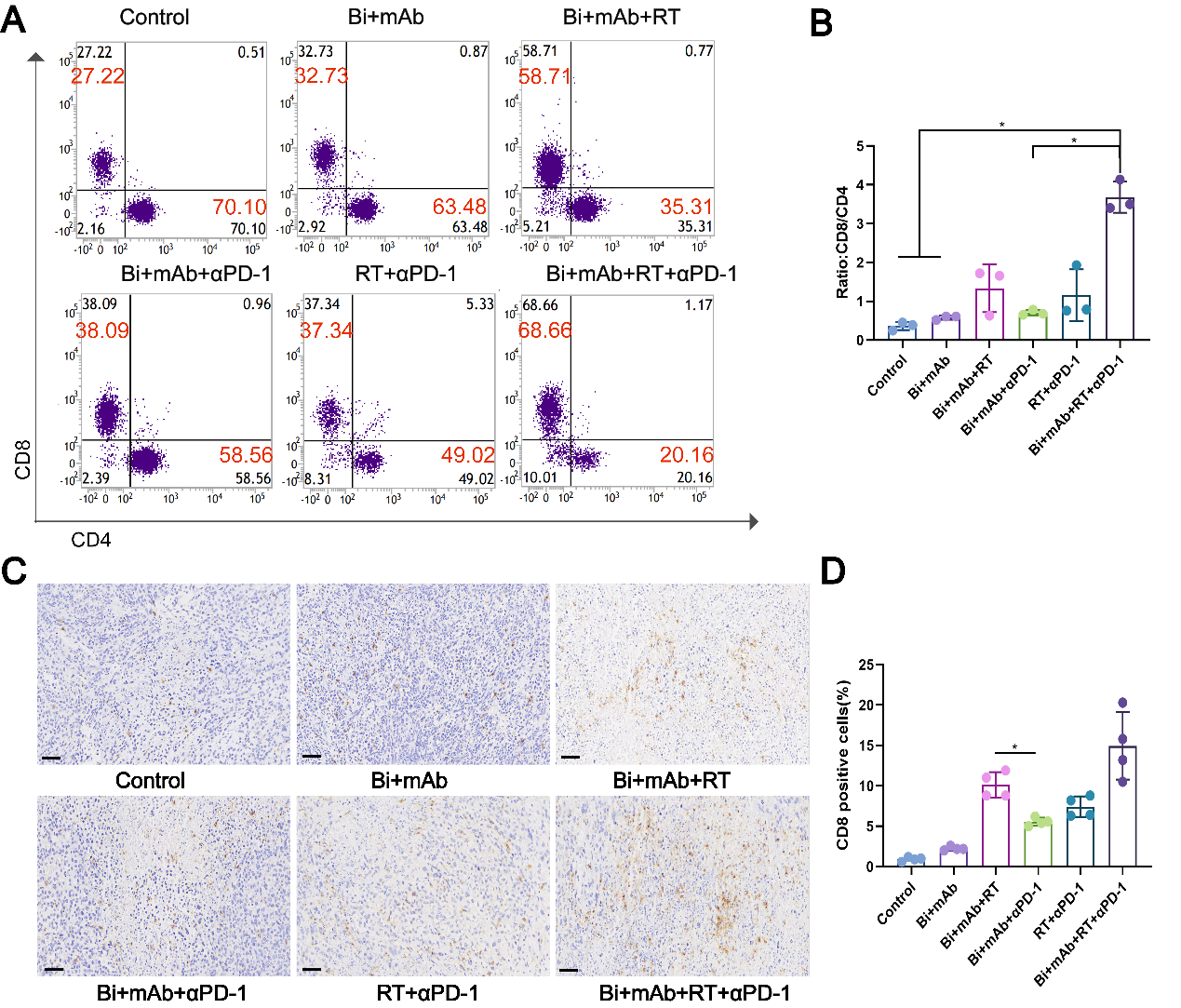


**Figure S6.** CD4+ and CD8+ T infiltration in primary tumors under different treatments. (A) Flow dot plots of T cells in primary tumors and (B) CD8+/CD4+ T ratio (n=3). (C) Representative images of CD8 immunohistochemistry in primary tumors and (D) percentage of CD8 expression (n=4). Scale bar, 50 μm. All data are presented as the means ± SD. Statistical analyses were performed by one-way ANOVA. ns: not statistically significant, **P<0.01.


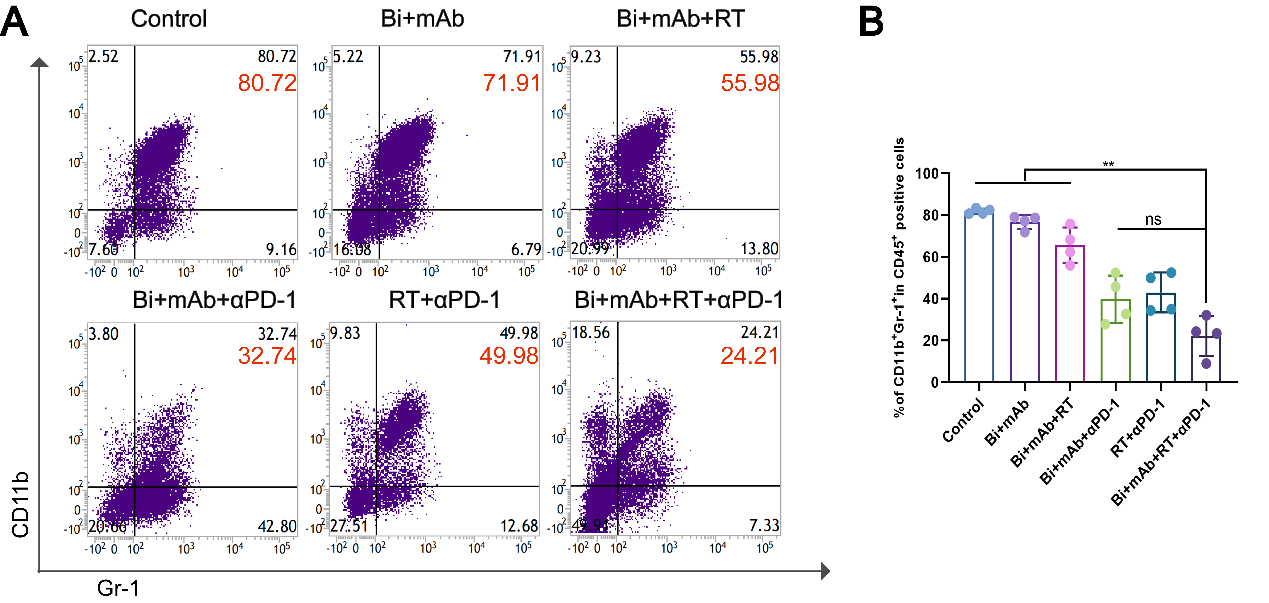


**Figure S7.** MDSC cell expression in primary tumors under different treatments. (A) Flow dot plots of MDSC cells in primary tumors and (B) percentages (n=4). All data are presented as the means ± SD. Statistical analyses were performed by one-way ANOVA. ns: not statistically significant, **P<0.01.


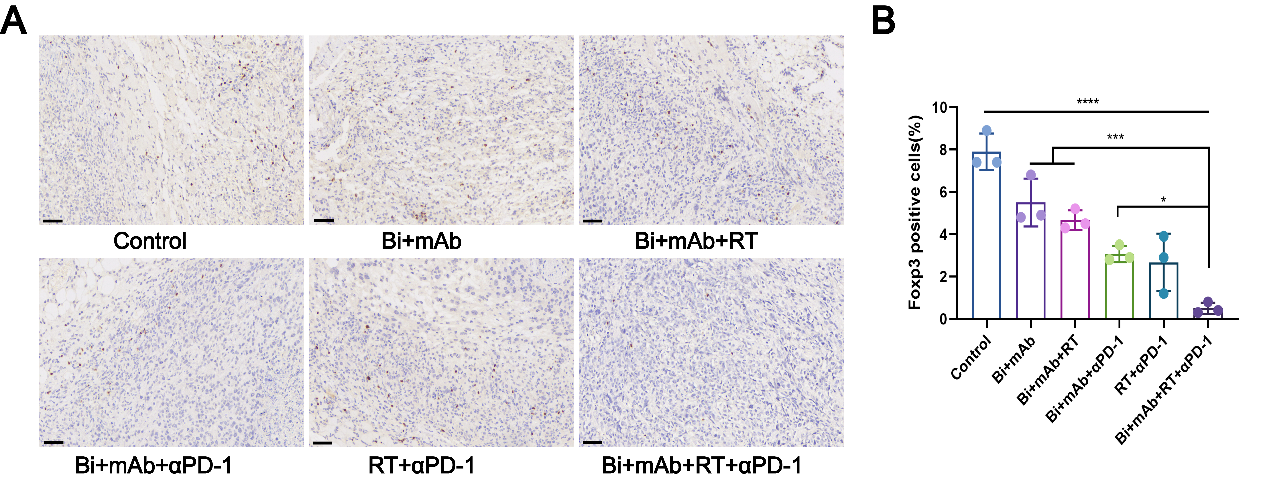


**Figure S8.** Treg cell expression in primary tumors under different treatments. (A) Representative images of Foxp3 immunohistochemistry in primary tumors and (B) percentage of Foxp3 expression (n=3). Scale bar, 50 μm. All data are presented as the means ± SD. Statistical analyses were performed by one-way ANOVA. *P<0.05, ***P<0.001, ****P<0.0001.


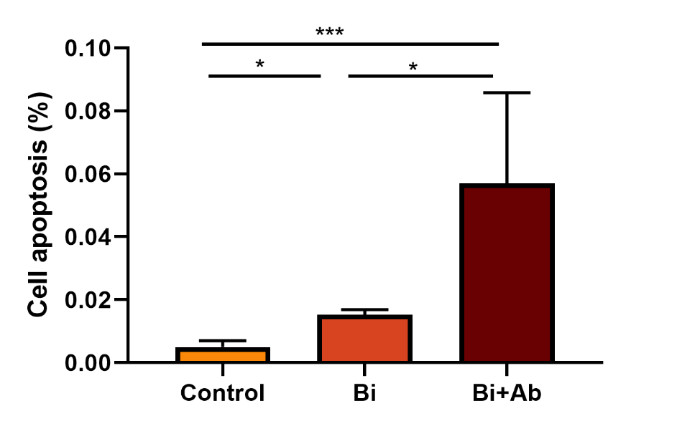


**Figure S9.** Percentage of apoptotic cells in tumors (n=3). All data are presented as the means ± SD. Statistical analyses were performed by one-way ANOVA. *P<0.05, ***P<0.001.


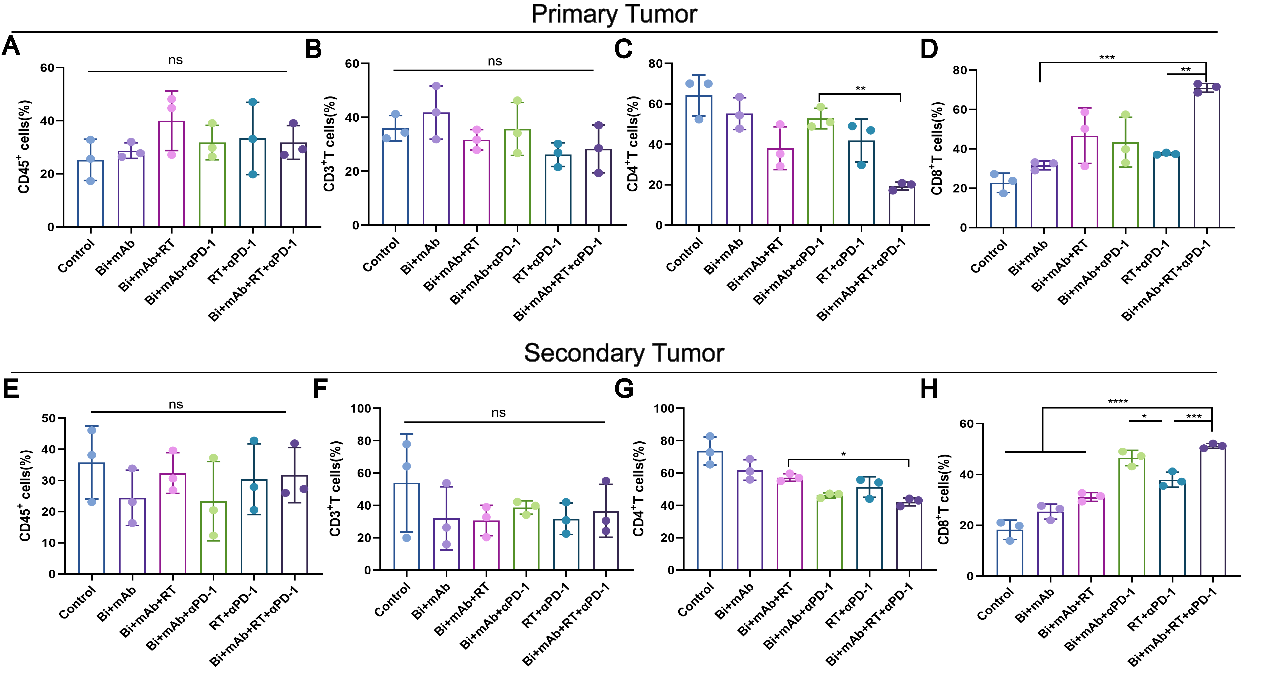


**Figure S10.** Infiltration of T cells in bilateral tumors. (A to D) Proportions of CD45+ cells (A), CD3+ T cells (B), CD4+ T cells (C), and CD8+ T cells (D) in primary tumors. (E to H) Proportions of CD45+ cells (E), CD3+ T cells (F), CD4+ T cells (G), and CD8+ T cells (H) in secondary tumors (n=3). All data are presented as the means ± SD. Statistical analyses were performed by one-way ANOVA. ns: not statistically significant, *P<0.05, **P<0.01, ***P<0.001, ****P<0.0001.
