# Supplementary material for: Antibody targeting of anaerobic bacteria warms cold tumors and improves the abscopal effect of radiotherapy
Source: J Transl Med. 2024 Jul 15;22:657. doi: 10.1186/s12967-024-05469-0 (PMC11247849; doi:10.1186/s12967-024-05469-0)
Supplement: Supplementary file 2 — Supplementary Material 2 [file 12967_2024_5469_MOESM2_ESM.docx]

**Request for change in authorship**

Dear Editor,

Hopefully it won't bother you, here we are hoping to make changes to authorship. We sent a "Request for change in authorship" email to editorialmanager, a few days ago, but unfortunately we didn't receive a reply. Although we have expressed our wish to increase the author contributions, the final decision is yours and we respect it and will follow your decision!

Reference:JTRM-D-24-01677

Article Entitled: Antibody targeting of anaerobic bacteria warms cold tumors and improves the abscopal effect of radiotherapy

**WeiZhou Wang^1#^, YunXue Zheng^1#^, ZhouXue Wu^5#^, Min Wu^1^, Yue Chen^2,4^, Yan Zhang^5*^, ShaoZhi Fu^1,2*^, JingBo Wu^1,2,3*^**

1. Department of Oncology, The Affiliated Hospital of Southwest Medical University, Luzhou, Sichuan, 646000, China
2. Nuclear Medicine and Molecular Imaging Key Laboratory of Sichuan Province, Luzhou, Sichuan, 646000, China
3. Academician (Expert) Workstation of Sichuan Province, Luzhou, Sichuan, 646000, China
4. Department of Nuclear Medicine, The Affiliated Hospital of Southwest Medical University, Luzhou, Sichuan, 646000, China
5. Department of Oncology and Hematology, Affiliated Hospital of Traditional Chinese Medicine of Southwest Medical University, Luzhou, China

\# WeiZhou Wang, YunXue Zheng, and ZhouXue Wu contributed equally to this work.

***Correspondence**:

JingBo Wu: [wjb6147@163.com;](mailto:wjb6147@163.com;) ShaoZhi Fu: [shaozhifu513@163.com;](mailto:shaozhifu513@163.com;) Yan Zhang: [113804562@qq.com](mailto:113804562@qq.com)

Because of the additional contributions to this paper and the support work provided by ZhouXue Wu and Yan Zhang, we need to change the current manuscript's author order and addresses.

Among the authors in the list, ZhouXue Wu was involved in preparing the figures and searching the literature. Yan Zhang provided support, supervision and review of the experimental environment.

After consultations, all the authors agreed with the addition of authors in this paper, and all the authors agreed with the rearrangement of the names.

We have no conflicts of interest to disclose.

Thank you for your consideration of this manuscript.

Sincerely,

Jingbo Wu, professor,

Corresponding author

Department of Oncology, the Affiliated hospital of Southwest Medical University, No.25 Taiping Street, Jiangyang District, Luzhou, Sichuan, 646000, China

**Cover letter**

Dear Editor,

We wish to submit a manuscript as an original research article entitled “**Antibody targeting of anaerobic bacteria warms cold tumors and improves the abscopal effect of radiotherapy**” for consideration by Journal of Translational Medicine.

# We confirm that this work is original and has not been published elsewhere, nor is it currently under consideration for publication elsewhere. The manuscript has been edited by a professional company (www.mjeditor.com). Thank you very much for taking time to review this newly manuscript.

# The anaerobic resistance of solid tumors to radiotherapy and their own "cold" state prevent the abscopal effects of RT combined with immunotherapy; therefore, new treatment options are needed to be explored to enhance this effect. *Bifidobacterium* (Bi) is an immunopotentiator for strict anaerobic survival. In this study, Bi was used as a tumor anaerobic targeting bullet to attract Bi-specific antibodies (mAb) in order to reach the tumor site through the antigen-antibody reaction. This mAb acted as a bacterial antibody and did not target tumor antigens, thereby disregarding the cancer's genotypic characteristics. Therefore, it can be used for the anaerobic-targeted therapy in a wide range of solid tumors. Our previous study demonstrated that the combined application of Bi+mAb could target and destroy the hypoxic regions of tumors and act as a radiosensitizer. In this study, we explored the anti-tumor efficacy of quadruple therapy (Bi+mAb + RT+αPD-1). This study also aimed to probe into the complex immune mechanisms underlying this phenomenon.

# In the present study, first, the activation of innate immune response was explored after the *in vivo* binding of Bi to mAb by promoting cellular and humoral immunity via killer cells and complement system, respectively. Second, the combination therapy upregulated the infiltration of mature DCs, increased antigen presentation, promoted CD8+ T cell infiltration in secondary tumors, and stimulated adaptive immune responses. Finally, quadruple therapy downregulated the expression of MDSCs and Treg cells in tumors, reversed the immunosuppressive microenvironment, and provided a favorable microenvironment for CD8+ T cells to kill tumors. Potentially, the present study provided a new idea for the pleiotropic immune-activating function of antibody-targeted anaerobic bacteria for the RT treatment of cancer patients with extensive metastases.

# We believe that this manuscript is appropriate for publication by Journal of Translational Medicine because it is related to microbial products and cancer immunotherapy.

We have no conflicts of interest to disclose.

Please address all correspondence concerning this manuscript to me at wjb6147@163.com.

Thank you for your consideration of this manuscript.

Sincerely,

Jingbo Wu, professor,

Corresponding author

Department of Oncology, the Affiliated hospital of Southwest Medical University, No.25 Taiping Street, Jiangyang District, Luzhou, Sichuan, 646000, China
